# *Nfkbie*-deficiency leads to increased susceptibility to develop B-cell lymphoproliferative disorders in aged mice

**DOI:** 10.1038/s41408-020-0305-6

**Published:** 2020-03-13

**Authors:** Veronique Della-Valle, Damien Roos-Weil, Laurianne Scourzic, Enguerran Mouly, Zakia Aid, Walaa Darwiche, Yann Lecluse, Frederik Damm, Sylvie Mémet, Thomas Mercher, Said Aoufouchi, Florence Nguyen-Khac, Olivier A. Bernard, Hussein Ghamlouch

**Affiliations:** 1 INSERM U1170; équipe labélisée Ligue Nationale Contre le Cancer; Gustave Roussy, Villejuif, France; 2 Faculté de Médecine, Université Paris-Sud, Université Paris-Saclay, Orsay, France; 3 Sorbonne Université, Service d’hématologie clinique, Hôpital Pitié-Salpêtrière, APHP, Paris, France; 4 INSERM U1138, Centre de Recherche des Cordeliers, Sorbonne Université, Paris, France; 5 EA4666 Lymphocyte Normal – Pathologique et Cancers, HEMATIM, Université de Picardie Jules Verne, and Laboratoire d’Hématologie, Centre Hospitalier Universitaire Amiens-Picardie, Amiens, France; 6 PFIC, Integrated Biology Core Facility, UMS 23/3655, Université Paris-Saclay, Gustave Roussy, Villejuif, France; 7 Charité-Universitätsmedizin Berlin, corporate member of Freie Universität Berlin, Humboldt-Universität zu Berlin, and Department of Hematology, Oncology, and Tumor Immunology, Berlin Institute of Health, Berlin, Germany; 8 Aix Marseille Univ, CNRS, INSERM, CIML, Centre d’Immunologie de Marseille-Luminy, Marseille, France; 9 CNRS UMR8200; équipe labélisée Ligue Nationale Contre le Cancer; Gustave Roussy, Université Paris-Saclay, Villejuif, France; 10 Service d’Hématologie Biologique, Sorbonne Université, Hôpital Pitié-Salpêtrière, APHP, Paris, France

**Keywords:** B-cell lymphoma, Chronic lymphocytic leukaemia, B cells, Oncogenes

## Abstract

Aberrant NF-κB activation is a hallmark of most B-cell malignancies. Recurrent inactivating somatic mutations in the *NFKBIE* gene, which encodes IκBε, an inhibitor of NF-κB-inducible activity, are reported in several B-cell malignancies with highest frequencies in chronic lymphocytic leukemia and primary mediastinal B-cell lymphoma, and account for a fraction of NF-κB pathway activation. The impact of *NFKBIE* deficiency on B-cell development and function remains, however, largely unknown. Here, we show that *Nfkbie*-deficient mice exhibit an amplification of marginal zone B cells and an expansion of B1 B-cell subsets. In germinal center (GC)-dependent immune response, *Nfkbie* deficiency triggers expansion of GC B-cells through increasing cell proliferation in a B-cell autonomous manner. We also show that *Nfkbie* deficiency results in hyperproliferation of a B1 B-cell subset and leads to increased NF-κB activation in these cells upon Toll-like receptor stimulation. *Nfkbie* deficiency cooperates with mutant MYD88 signaling and enhances B-cell proliferation in vitro. In aged mice, *Nfkbie* absence drives the development of an oligoclonal indolent B-cell lymphoproliferative disorders, resembling monoclonal B-cell lymphocytosis. Collectively, these findings shed light on an essential role of IκBε in finely tuning B-cell development and function.

## Introduction

The NF-κB signaling pathway plays essential roles in cell survival, differentiation, proliferation, inflammation, and immune regulation^1^. Aberrant NF-κB activation is frequently observed in several B-cell malignancies^2–6^. This activation can result from interactions with the tumor microenvironment^7–9^ or as a result of somatic mutations affecting NF-κB family members or upstream signaling components, such as CD79B, BIRC3, CARD11, MYD88, TNFAIP3/A20, or inhibitors of kappa B (IκBs; refs. ^3,5,6,10–13^). The inactivation of IκB genes is recurrently observed in human B-cell malignancies. The NF-κB inhibitor I-kappa-B-epsilon (*NFKBIE*) gene, which encodes IκBε, is targeted by a recurrent 4-bp truncating mutation in addition to rare point mutations in 5–10% of chronic lymphocytic leukemia (CLL)^2,3,14–16^ and various B-cell lymphomas (BCLs), including diffuse large B-cell lymphoma (DLBCL; 5%), mantle cell lymphoma (5%), splenic marginal zone lymphoma (1.8%), and primary mediastinal B-cell lymphoma (PMBCL; 23% of patients)^12,17^. *NFKBIE* mutations are enriched among advanced stage CLL and associated with poor-prognostic outcome, suggesting that they might be involved in disease progression^2,3,12,17^. Compared to NFKBIE-wild-type (WT) patients, *NFKBIE*-mutated CLL cells showed reduced IκBε protein levels and decreased p65 inhibition, along with increased phosphorylation and nuclear translocation of p65 (ref. ^2^).

The NF-κB transcription factor family members are maintained as inactive homo- or heterodimers in the cytoplasm by IκB, including the classical IκB protein family: IκBα, IκBβ and IκBε (ref. ^1^). IκBε is an inhibitor of inducible NF-κB activity, which traps the Rel proteins in the cytoplasm at the resting state^1,18,19^. Its expression is higher in primary murine splenic B cells than in T cells and has a specific role in the late retro-control of B-cell response to external stimuli, including signaling through the B-cell receptor (BCR) and Toll-like receptors (TLRs)^20–22^. IκBε is more expressed in IgM+ B cells than in IgG+ B cells and presents a slower turnover than IκBα and IκBβ, following in vitro activation of primary splenic B cells with LPS or a combination of anti-IgM and CD40L (ref. ^21^). IκBε shows preferential specificity for NF-κB Rel homodimers, with respect to other NF-κB transcription factor subunits^21^. IκBε-deficient B cells present higher expression and activity of Rel at early time points of activation with IgM and IL4 compared to WT cells^20–22^.

To get more insights into the role of *NFKBIE* in normal and malignant B-cell differentiation, we studied *Nfkbie*-deficient mice and showed that the loss of *Nfkbie* results in marginal zone B (MZB) and B1 cells expansion, and a higher sensitivity to T-cell-dependent and -independent stimulation. We also show that *Nfkbie* deficiency cooperates with mutant MYD88 signaling and causes enhanced B-cell proliferation. In aged mice, *Nfkbie* absence drives development of an oligoclonal indolent B-cell lymphoproliferative disorders, resembling monoclonal B-cell lymphocytosis (MBL).

## Materials and methods

Additional information can be found in the Supplemental Methods.

### Mice

Inactivated *Nfkbie* allele on a mixed Sv129xDBA-2xC57BL/6 J background has been described previously;^23^ >20 back-crosses were performed on the C57BL/6 J background to give rise to a pure congenic *Nfkbie*^*−/*^^−^ C57BL/6 J strain. Mutant mice in this pure C57BL/6 J background were housed in the animal facility of the Gustave Roussy Institute (SCEA, Gustave Roussy, Villejuif, France), crossed with WT C57BL/6 J mice and age-matched animals of selected genotypes were sacrificed at the indicated times. Bone marrow transplantation were performed as described^24,25^. Animal experiments were conducted in compliance with the Gustave Roussy Institutional guidelines and authorized by the Direction Départementale des Services Vétérinaires du Val de Marne.

### Mice immunization

For the analysis of germinal center (GC) formation in T-cell-dependent immune response, *Nfkbie*-deficient and WT age-matched 2–3-month-old mice were immunized with sheep red blood cells (SRBCs; 1 × 10^8^ cells per mouse) via intraperitoneal injection. Mice were sacrificed 10 days after immunization and spleens were analyzed for GC B cells and plasma cells.

For in vivo TLR9 activation, CpG ODN (Invivogen) was administered intraperitoneally at a dose of 333 μg per mouse in 200 μL phosphate-buffered saline. Mice were sacrificed 10 days after immunization; inguinal lymph nodes and spleens were analyzed for GC B cells.

### In vitro generation of induced GC B cells

Splenic untouched resting murine B cells were first isolated from 2 to 3-month-old animals using the mouse CD43 (Ly-48) B-cell isolation kit (MicroBeads, Miltenyi Biotec) according to the manufacturer’s instructions (yield > 90% CD19+B220+). MZB and FOB cells were flow-sorted as described in the “Cell isolation and culture” section. 40LB-cells expressing CD40L and producing BAFF were a kind gift of Pr. D. Kitamura^26^ and cultured in DMEM media (Invitrogen) with 10% FBS and penicillin/streptomycin.

For B-cell culture, RPMI-1640 medium (Invitrogen) was supplemented with 10% FBS, 0,055 mM 2-ME, 10 mM HEPES, 1 mM sodium pyruvate, 100 units/ml penicillin, and 100 μg/ml streptomycin (invitrogen). Purified B cells (110 × 10^3^ cells per well) were cultured in a six-well plate in the presence of 40LB-cells (0.5 × 10^6^ cells per dish) that had been irradiated with 80 Gy X-ray. rIL-4 (1 ng/ml; Peprotech) was added to the primary culture for 4 days. Cells were harvested at day 4 (D4) and processed for flow cytometric analysis.

### MBL diagnosis

The appearance of a B220lowCD19+CD5+ population and its percentage among peripheral blood mononuclear cells (PBMCs) were used for initial MBL-like disease diagnosis. The diagnostic criterion for MBL in mice was arbitrary defined as the appearance of oligoclonal/monoclonal B220lowCD19+CD5+ cells, constituting over 5% of PBMCs without a detectable increase in total white blood cell count.

### Genescan analysis

cDNA from sorted *Nfkbie*-deficient B cells (B220lowCD19+) and WT B cells (B220+CD19+) was PCR amplified using VH family primers (VH1, VH2, VH3, VH5, VH6, and VH7 as indicated) and a common JH FAM-conjugated primer to assess IgH CDR3 diversity^27^. PCR products were run on a 3130xl sequencer (Applied Biosystems) and data were analyzed using the Peakscanner software (Applied Biosystems).

### Retroviral infection and in vivo cell transfer

MYD88WT and that containing L265P mutation (MYD88L265P) cDNA were synthesized by GenScript and subcloned into MSCV-eGFP backbone. Viral particles and transduction procedures were described previously^3^.

### Statistical analysis

Statistical significance of differences between the results was assessed using a two-tailed unpaired Student’s *t*-test with Welch’s correction, performed using Prism (GraphPad software, version 5.03). Statistically significant *p* values: *p values < 0.05; **p values < 0.01 and ***p values < 0.005. Error bars displayed throughout the paper represent s.e.m. or s.d. as indicated in figure legends. No statistical method was used to predetermine sample size. No blinding and no randomization of samples were applied. No data was excluded.

## Results

### *Nfkbie*^−/−^ mice show amplification of MZB and B1 B cells

**Fig. 1:**
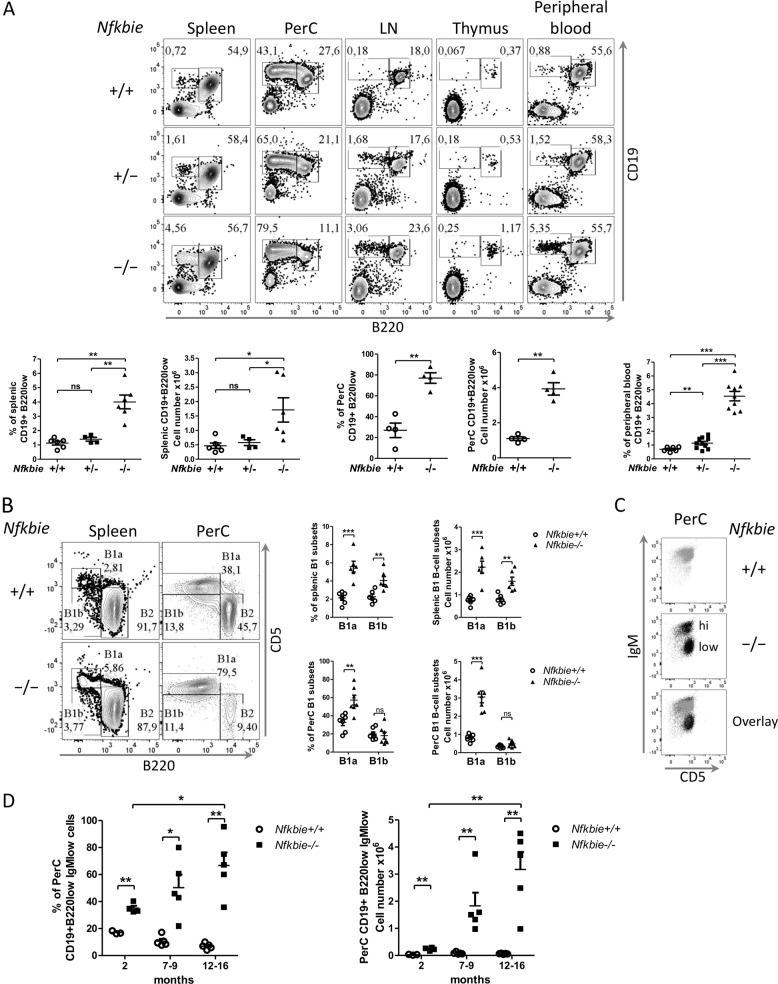
*Nfkbie*^**−**/−^ mice present an expansion of B1a B cells and a biased differentiation pathway toward MZB. **a** Upper panel: representative plots of FACS analysis of the CD19+B220low population in spleen, peritoneal cavity (PerC), lymph node (LN), thymus, and peripheral blood of *Nfkbie*^+/+^, *Nfkbie*^+/−^, and *Nfkbie*^**−**/−^ mice. Lower panel: percentages and absolute cell numbers of the CD19+B220low population in spleen and PerC, and percentages of the CD19+B220low population in peripheral blood. Each symbol represents one mouse. **b** Analysis of the B1 B-cell subset, B1a (CD19+B220lowCD5+) and B1b (CD19+B220lowCD5−), partitioning in spleen and PerC of *Nfkbie*^+/+^ and *Nfkbie*^**−**/−^ mice (*n* = 5). Each symbol represents one mouse. **c** Representative FACS plots of IgM and CD5 expression on the CD19+B220low cells in PerC of *Nfkbie*^+/+^ (*n* = 5) and *Nfkbie*^**−**/−^ mice (*n* = 5). **d** Percentages and absolute cell numbers of CD19+B220low IgMlow B cells in the PerC of *Nfkbie*^+/+^, and *Nfkbie*^**−**/−^ of 2, 7–9 and 12–16-month-old mice (*n* = 5). Each symbol represents one mouse. Data are mean ± SEM. *p < 0.05, **p < 0.01, ***p < 0.001; ns, not significant.

Initial analyses of immune cells in *Nfkbie*^−/−^ mice on a mixed genetic background showed only very subtle phenotypic consequences on thymic lymphoid T-cell subsets^23^. Our analysis showed that, compared to 5–8-month-old WT, aged-matched *Nfkbie*^−/−^ mice on a pure C57BL/6 J background presented an increase in cell number and frequency of a population expressing low levels of the B220 marker (B220low) in spleen, thymus, lymph nodes, peritoneal cavity, and peripheral blood (Fig. 1a). Detailed immunophenotyping in spleen and peritoneal cavity was compatible with a B1 B-cell population: CD19+, B220low/−, CD43+, CD23−, CD21−, CD93−, IgDlow, and IgM+ (Supplementary Fig. 1a).

Analysis of CD5 expression showed that both B1a (CD5+) and B1b (CD5−) subsets were increased in spleen of *Nfkbie*^**−**/−^ mice, whereas only the B1a cell population augmented in peritoneal cavity (Fig. 1b). Surface IgM expression on B1 cells was higher in *Nfkbie*^**−**/−^ than in WT splenic cells, in contrast to peritoneal ones (Supplementary Fig. 1b). Based on their surface IgM expression levels, peritoneal cavity *Nfkbie*^**−**/−^ (B220lowCD19+CD5+) B1a cells were subdivided into IgMhigh and IgMlow B1a subsets (Fig. 1c). The IgMlow B1a B-cell population increased with age in *Nfkbie*^**−**/−^ mice (Fig. 1d).

B2 populations flow cytometry analysis revealed lower frequency and numbers of mature follicular B (FoB) cells, defined as CD19+B220+CD23+CD21+, and increased frequency and numbers of MZB cells, defined as CD19+B220+CD23lowCD21hi, in *Nfkbie*^**−**/−^ mice with respect to WT (Supplementary Fig. 1d), as reported^20^. *Nfkbie*^**−**/−^ MZB cells displayed significantly higher IgM expression than WT MZB cells, unlike FoB cells WT and *Nfkbie*^**−**/−^ cells (Supplementary Fig. 1c).

Together, these data indicate that the absence of *Nfkbie* affects mature B-cell subsets differentiation and leads to expansion of MZB and B1a B cells. These B-cell subsets are known to mediate the innate functions of the B lineage. Both populations are particularly sensitive to variations in NF-κB activity and strongly influenced by BCR specificity and strength of signaling^28–30^.

### *Nfkbie* deficiency affects the frequency of the B1 B-cell progenitor and the transition from transitional B cells to mature B cells

We next analyzed in detail hematopoietic differentiation, including B-cell development, in the bone marrow of 2-month-old KO mice. Proportions of LSK cells, myeloid (CMP, GMP, and MEP), and common lymphoid progenitor were comparable between WT, *Nfkbie*^+/−^ and *Nfkbie*^**−**/−^ mice (Supplementary Fig. 2a–c). Analysis of early B-cell differentiation stages, including pre-pro-B (CD19−B220+CD43+), pre-B (CD19+B220+CD43−), and pro-B (CD19+B220+CD43+), showed no significant alteration of the percentages and numbers of each population (Supplementary Fig. 2d, f). Analysis of Hardy fractions did not reveal any abnormalities in *Nfkbie*^**−**/−^ mice either (Supplementary Fig. 2e, g), indicating that loss of *Nfkbie* does not impact early B-cell development in the bone marrow.

**Fig. 2:**
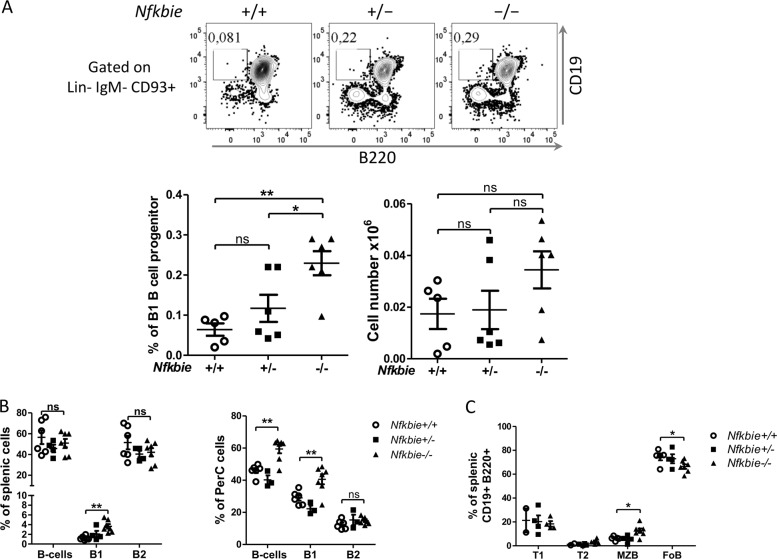
*Nfkbie*^**−**/−^ deficiency alters the frequency of B1 progenitor cells and the transition from transitional to mature B cells. **a** Bone marrow B1 B-cell progenitor analysis of 2-month-old mice. Upper panel: representative plots of FACS analysis of B1 (Lin−IgM−CD93+B220−CD19+) B-cell progenitor. Lower panel: percentage and absolute cell numbers of the bone marrow Lin−IgM−CD93+B220−CD19+ population. Each symbol represents one mouse. *Nfkbie*^+/+^ (*n* = 5), *Nfkbie*^+/−^ mice (*n* = 6) and *Nfkbie*^**−**/−^ mice (*n* = 6). **b** Analysis of total B cells (CD19+), B1 (CD19+B220low) and B2 (CD19+B220+) subset distribution in the spleen and peritoneal cavity. Each symbol represents one mouse. *Nfkbie*^+/+^ (*n* = 5), *Nfkbie*^+/−^ mice (*n* = 6), and *Nfkbie*^**−**/−^ mice (*n* = 6). **c** Analysis of splenic transitional (T1 (CD19+B220+CD93+IgMhiCD23−) and T2 (CD19+B220+CD93+IgMhiCD23+)) B cells, MZB cells and FoB cells distribution. Each symbol represents one mouse. *Nfkbie*^+/+^ (*n* = 5), *Nfkbie*^+/−^ mice (*n* = 6), and *Nfkbie*^**−**/−^ mice (*n* = 6). Data are mean ± SEM. *p < 0.05, **p < 0.01; ns, not significant.

We then evaluated the frequency and numbers of B1 B-cell progenitors (Lin−CD93+CD19+B220-/low) in mutant bone marrow^31^. Significantly higher frequency of Lin−CD93+CD19+B220−/low cells in 2-month-old *Nfkbie*^**−**/−^ mice were observed compared to those of WT or *Nfkbie*^+/−^ mice, with a trend for *Nfkbie*^**−**/−^ B1 progenitor cell number increases (Fig. 2a).

To check the balance between B1 and B2 cells, we analyzed their proportions in the spleen and peritoneal cavity. Percentages of total CD19+ B cells increased significantly in peritoneal cavity but remained stable in the spleen of *Nfkbie*^**−**/−^ mice, as compared to WT animals (Fig. 2b). Percentages of splenic and peritoneal B2 cells (CD19+B220+) were also similar between both genotypes (Fig. 2b); however, percentages of B1 cells were significantly higher in *Nfkbie*^**−**/−^ mice. These data indicate that there is no shift toward the B1 cell lineage in mutant mice and that the increase in B1 cell numbers might result from the increase of the B1 B-cell progenitor cells.

We next analyzed peripheral splenic B2 B-cell populations from 2-month-old mutant mice. Immature transitional B-cells frequencies and numbers were unaffected by *Nfkbie* deficiency, whereas decrease of mature FoB-cell population and increase of MZB cells observed in older mice were already present (Fig. 2c). Additional analyses of non-B-cell lineage did not show the reported CD44− CD25+(DN3) thymocytes decrease (Supplementary Fig. 2h), which might therefore result from the mixed genetic background of the mutant mice^23^. No other abnormality of major hematopoietic lineages was observed in 2-month-old mice (Supplementary Fig. 2i) or older *Nfkbie*^**−**/−^ (Supplementary Fig. 2j) mice.

To assess whether higher B1 and MZB B-cell numbers were due to increased proliferation or reduced apoptosis, we investigated ex vivo proliferation and apoptosis. No significant differences in the proportion of Ki67+ or viable MZB, FoB, B1 B cells between *Nfkbie*^**−**/−^ and WT mice were detected (Supplementary Fig. 3a, b). As the frequency and cell numbers of splenic transitional B cells were equivalent between *Nfkbie*^**−**/−^ and WT mice, these findings suggest that *Nfkbie* deficiency biases the differentiation of transitional B cell into MZB cell fate.

Overall, these data indicate that *Nfkbie* is important for follicular versus MZB cell fate decision and that its loss may affect the size of the B1 B-cell progenitor compartment.

### Biased differentiation toward MZB cell and expansion of B1 B-cell subsets in absence of *Nfkbie* is cell-autonomous

**Fig. 3:**
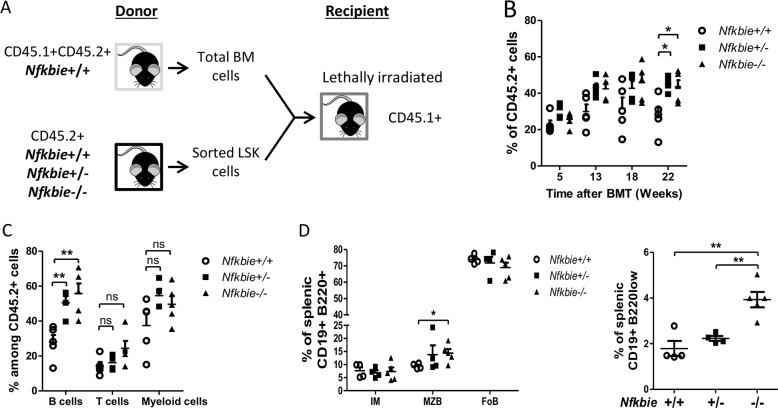
Biased differentiation of mature B-cell subsets in absence of *Nfkbie* is cell-autonomous. **a** Scheme of the competitive BM reconstitution assay. **b** Percentage of CD45.2+ (donor cells) in peripheral blood of CD45.1 recipient chimeric mice along time after adoptive transfer (*n* = 5). **c** Percentage of CD45.2+ B, T, and myeloid cells in the spleen of CD45.1 recipient chimeric mice 7 months post transfer. Each symbol represents one mouse (*n* = 5). **d** Analysis of peripheral splenic CD45.2 donor B-cell populations in recipient chimeric mice 7 months post transfer. Left panel: follicular (FoB: CD23+CD21+), marginal zone (MZB: CD23lowCD21high), and immature B cells (IM: CD23−CD21−) distribution. Right panel: splenic B1 B-cell percentage. Each symbol represents one mouse (*n* = 5). Data are mean ± SEM. *p < 0.05, **p < 0.01; ns, not significant.

To investigate whether *Nfkbie* deficiency-associated changes were cell-autonomous, we performed competitive bone marrow reconstitutions (Fig. 3a for scheme). FACS analysis in peripheral blood showed that recipients of WT CD45.2+ cell had a stable reconstitution with ~30% donor cells, whereas there was a steady increase in the percentage of donor cells in recipients of *Nfkbie*^+/−^ and *Nfkbie*^−/−^ CD45.2+ cells that reached statistical significance at 22 weeks with over 40% of donor cells (Fig. 3b).

Analysis of the distribution of cell lineages showed that *Nfkbie*^+/−^ and *Nfkbie*^−/−^ LSK (Lin−Sca1+Kit+) cell transfer resulted in a greater proportion of CD19+ B cells than that of WT cells in recipient mice (Fig. 3c). Consistent with our initial observations, at 22 weeks *Nfkbie*^−/−^ cells contributed to a significantly higher proportion of splenic MZB cells and B220lowCD19+ B1 cells than WT ones (Fig. 3d). These data establish the cell-autonomous nature of the *Nfkbie*-deficient-associated phenotypes.

### Aged *Nfkbie*^−/−^ mice develop a MBL-like phenotype

**Fig. 4:**
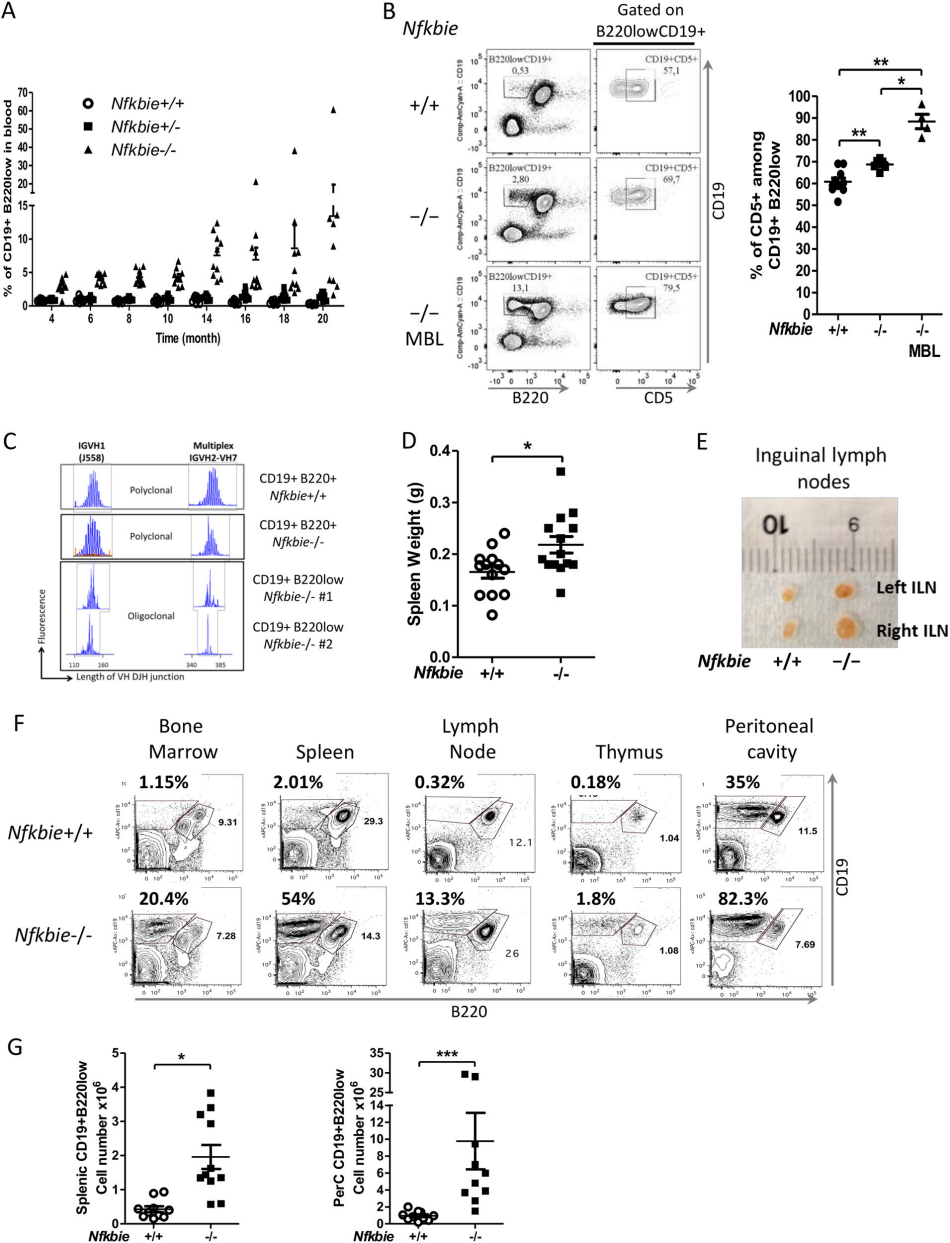
Aged *Nfkbie*^**−**/−^ mice develop a MBL-like phenotype. **a** Peripheral blood was monitored for the presence of CD19+B220low B cells starting at 4 months. Each symbol represents one mouse (*n* = 10). **b** Right panel: percentage of CD5+ cells among CD19+B220low population in the peripheral blood of 16-month-old mice. Each symbol represents one mouse. Left panel: representative plots of FACS analysis of the CD19+B220low B cells and of the expression of CD5 on the CD19+B220low population in the peripheral blood of *Nfkbie*^+/+^ (*n* = 10), *Nfkbie*^−/−^ without MBL(−/−) (*n* = 6), and *Nfkbie*^−/−^ with MBL (−/−MBL) (*n* = 4). **c** Analyses of BCR rearrangement clonality in sorted *Nfkbie*^+/+^ CD19+B220+ B cells, and *Nfkbie*^−/−^ CD19+B220+ and CD19+B220low B cells of two different 14-month-old mice (#1 and #2). V–J junctions were PCR amplified from cDNA of sorted B cells using VH family primers (VH1 and multiplexed PCR of VH2, VH3, VH5, VH6, and VH7) and FAM-conjugated consensus JH primer to assess the IgH CDR3 diversity. **d** Spleen weight of old (>12 months) *Nfkbie*^+/+^ (*n* = 13) and *Nfkbie*^−/−^ mice (*n* = 14). Each symbol represents one mouse. **e** Representative image from littermate *Nfkbie*^+/+^ and *Nfkbie*^−/−^ old mice showing enlarged inguinal lymph nodes of *Nfkbie*^−/−^ mice. **f** Representative flow cytometry profiles of CD19+B220low infiltration in bone marrow, spleen, lymph nodes, thymus, and peritoneal cavity of 18-month-old *Nfkbie*^−/−^ mice with MBL (*n* = 5) and age-matched *Nfkbie*^+/+^ mice. **g** Absolute cell numbers of CD19+B220low B cells in the spleen and peritoneal cavity of old *Nfkbie*^+/+^ and *Nfkbie*^−/−^ mice (*n* = 11). Each symbol represents one mouse. Data are mean ± SEM. *p < 0.05, **p < 0.01.

To assess the long-term impact of *Nfkbie* deficiency, we monitored monthly a cohort of ten *Nfkbie*^−/−^, ten *Nfkbie*^+/−^ mice, and ten control WT mice for the presence of the B220lowCD19+ population in blood. After 12 months, five out of ten (50%) *Nfkbie*^−/−^ mice started to develop MBL (defined as over 5% of B220lowCD19+ within PBMCs; Fig. 4a). Cytological analysis of blood smears from MBL developing *Nfkbie*^−/−^ mice showed larger lymphocytes with more abundant cytoplasm around the nucleus (Supplementary Fig. 4a). *Nfkbie*^−/−^ mice that developed MBL-like disease showed a clear expansion of CD5+ cells among the B220lowCD19+ population (Fig. 4b). Analysis of IGH gene rearrangements revealed an oligoclonal pattern of the B220lowCD19+ population in Nfkbie^−/−^ mice (Fig. 4c). However, total white blood cell counts were not significantly elevated in 20-month-old *Nfkbie*^−/−^ mice compared with those of control mice (Supplementary Fig. 4b) neither was survival rate of these aged mutant animals (not shown). A single *Nfkbie*^−/−^ mouse, devoid of MBL, developed a disseminated tumor in subcutaneous tissues in the cervical area, resulting in a swollen neck. Flow cytometric analysis of tumor-infiltrating cells showed a dominant population of B220+CD19+CD5− B-cell population (data not shown). Nevertheless, old *Nfkbie*^−/−^ mice (>12 months) showed a mild splenomegaly (Fig. 4d) and 60% (6/10) of mice presented enlarged lymph nodes (Fig. 4e). They had no systemic symptoms and flow cytometry, and cell count analysis revealed significant multiorgan infiltration of B220lowCD19+ cells (Fig. 4f, g).

### *Nfkbie*-deficient B1a cells hyper-proliferate in response to TLR stimulation and exhibit enhanced NF-κB signaling

**Fig. 5:**
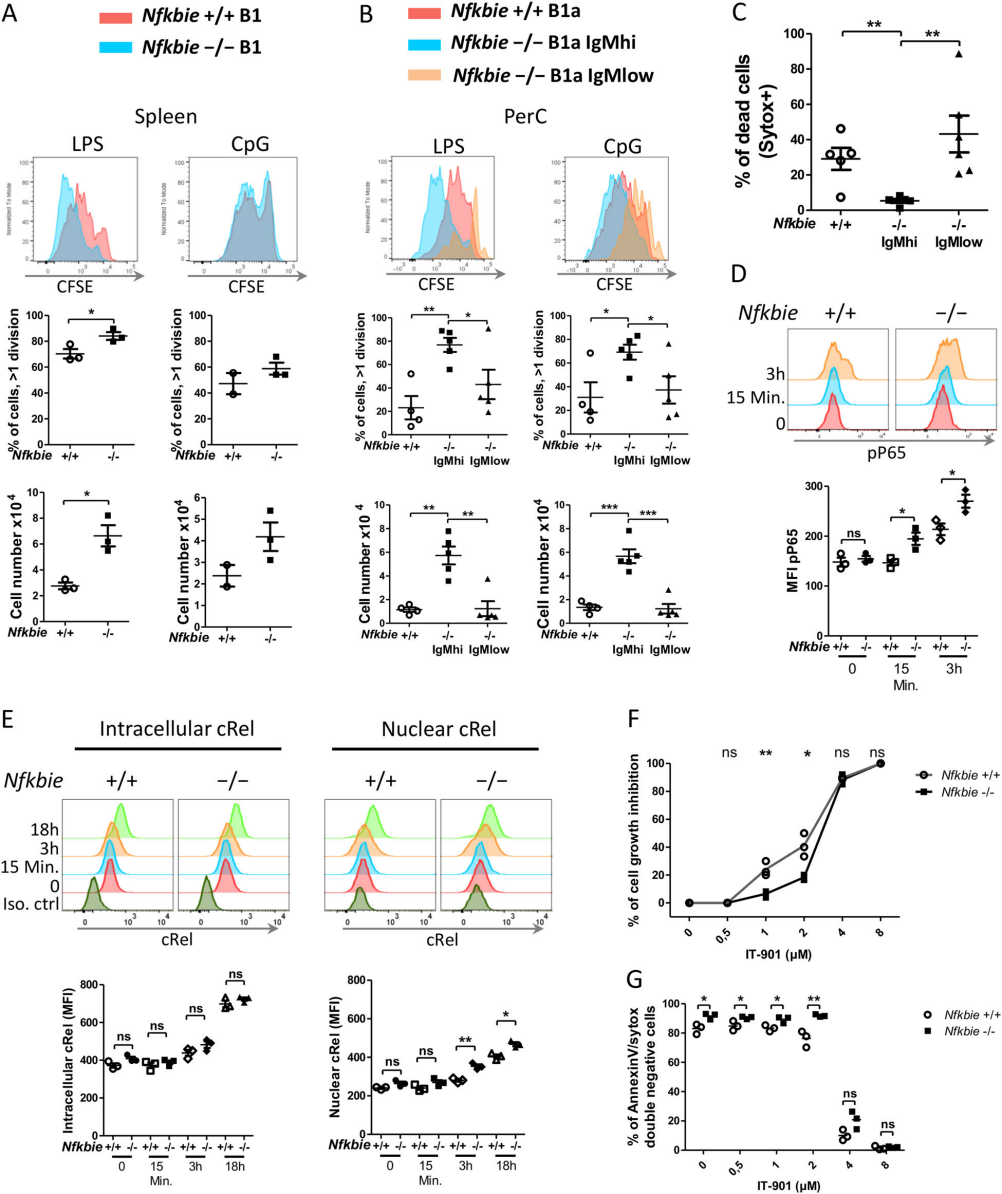
In B cells, IκBε sets the proliferation threshold in response to TLR or T-dependent antigen stimulations. **a** Sorted splenic B1 cells were labeled with CFSE and cultured in the presence of LPS or CpG. Cell division was measured by CFSE dilution at 72 h. Percentages of cells that underwent at least one division and cell numbers at 72 h are shown. Each symbol represents one mouse (*n* = 3). **b** Sorted peritoneal *Nfkbie*^+/+^ B1a, *Nfkbie*^−/−^ B1a IgMhi, and *Nfkbie*^−/−^ B1a IgMlow cells were labeled with CFSE, cultured in the presence of LPS or CpG, and analyzed as in **a**. Each symbol represents one mouse. *Nfkbie*^+/+^ (*n* = 4), *Nfkbie*^+/−^ mice (*n* = 5), and *Nfkbie*^−/−^ mice (*n* = 5). **c** Sorted peritoneal *Nfkbie*^+/+^ B1a, *Nfkbie*^−/−^ B1a IgMhi, and *Nfkbie*^−/−^ B1a IgMlow cells were cultured in complete medium without any stimulant for 24 h. Spontaneous cell death was assessed by sytox staining. Each symbol represents one mouse. *Nfkbie*^+/+^ (*n* = 4), *Nfkbie*^+/−^ mice (*n* = 5), and *Nfkbie*^−/−^ mice (*n* = 5). **d** Analysis of phosphorylated p65 (Ser536) in sorted *Nfkbie*^−/−^ and *Nfkbie*^+/+^ B1a B cells at T0 and after treatment with LPS for 15 min and 3 h. Each symbol represents one mouse (*n* = 3). **e** Analysis of intracellular and nuclear localization of cRel in sorted B1a *Nfkbie*^−/−^ and *Nfkbie*^+/+^ B cells at T0 and after treatment with LPS for 15 min, 3 h, and 18 h. To show nuclear localization of cRel, cytoplasm was removed from cRel-stained cells with nuclear isolation media (HBSS media with 1 mM EDTA and 0.5% NP-40). Each symbol represents one mouse (*n* = 3). **f** Sorted *Nfkbie*^−/−^ and *Nfkbie*^+/+^ B1a B cells were stimulated with LPS and treated for 72 h with serial dilutions of IT-901. At 72 h viable cell number was determined by cell count and trypan blue exclusion assay. The percentage of inhibition was calculated relative to vehicle-treated cells number. 0 stands for vehicle (DMSO). Each symbol represents one mouse (*n* = 3). **g** Sorted *Nfkbie*^−/−^ and *Nfkbie*^+/+^ B1a B cells were stimulated with LPS and treated for 72 h with serial dilutions of IT-901. Apoptosis was evaluated by Annexin V–PE and sytox staining. Percentages of double-negative (i.e., Annexin V-negative and sytox-negative) living cells are shown. 0 stands for vehicle (DMSO). Each symbol represents one mouse (*n* = 3). Data are mean ± SEM. *p < 0.05, **p < 0.01, ***p < 0.001.

We then investigated the impact of *Nfkbie* deficiency on the proliferative response of splenic and peritoneal B-cell subsets to T-cell independent stimuli, such as TLR agonists. These stimuli are known to induce NF-κB activity in B cells^1,8,20–22,32^. FACS-sorted splenic B-cell subsets, FoB (CD19+B220+CD23+CD21+), MZB (CD19+B220+CD23lowCD21hi), and B1 (CD19+B220low) cells were stimulated with anti-IgM, LPS, or CpG oligodeoxynucleotides, and cell division was measured by CFSE dilution and cell count after 72 h of culture. We found that splenic B1 (Fig. 5a) and MZB (Supplementary Fig. 5a) cells lacking *Nfkbie* displayed increased proliferation rate in response to LPS and CpG compared with WT B cells. Furthermore, *Nfkbie*^−/−^ MZB cells showed significantly higher proliferation rate in response to IgM stimulation compared to WT MZB cells (Supplementary Fig. 5a). There was no significant difference in FoB cells proliferation in response to LPS, CpG, and anti-IgM between mutant and WT cells (Supplementary Fig. 5b). As WT B1 cells, *Nfkbie*^−/−^ B1 cells did not respond to anti-IgM stimulation (data not shown). This confirms that MZB and B1 and to a lesser extent FoB cells require IκBε to limit their proliferative response in a B-cell-intrinsic manner.

We next focused our analysis on B-cell subsets from the peritoneal cavity. Peritoneal B2 B cells were sorted as CD19+B220+. Similarly to splenic FoB cells, increased proliferation of *Nfkbie*^−/−^ peritoneal B2 B-cells in response to stimuli did not reached statistical significance when compared with WT cells (Supplementary Fig. 5c).

In *Nfkbie*^−/−^ mice, peritoneal B1a B cells were sorted as two populations, B1a IgMhi and B1a IgMlow, while in *Nfkbie*^+/+^ mice B1a B cells were sorted as one single population expressing high levels of IgM (Fig. 1c). Analysis of IgM expression levels between *Nfkbie*^+/+^ B1a cells, *Nfkbie*^−/−^ B1a IgMhi showed no significant difference (Supplementary Fig. 5d). Similarly to WT cells, *Nfkbie*^−/−^ B1a IgMhi and IgMlow cells did not respond to anti-IgM stimulation (data not shown). *Nfkbie*^−/−^ B1a IgMhi cells showed higher proliferation in response to LPS or CpG stimulation compared to WT B1a B cells (Fig. 5b), whereas *Nfkbie*^−/−^ B1a IgMlow cell response was similar to that of WT B1a B cells and significantly inferior to that of *Nfkbie*^−/−^ B1a IgMhi (Fig. 5b).

Spontaneous cell death assessment of sorted WT B1a cells, *Nfkbie*^−/−^ B1a IgMhi and IgMlow subsets was quantified after 24 h ex vivo culture without any stimuli using sytox blue staining. A minor fraction of *Nfkbie*^−/−^ B1a IgMhi cells underwent cell death in such ex vivo culture (Fig. 5c). However, WT B1a cells and *Nfkbie*^−/−^ B1a IgMlow cells showed a significant higher death ratio than *Nfkbie*^−/−^ B1a IgMhi cells (Fig. 5c).

We next sought to investigate how IκBε deficiency impacts NF-κB activation in CD5+B1a B cells through analyzing phosphorylated p65 (RelA) levels and cRel nuclear localization by flow cytometry^33,34^. We found significantly higher levels of p65 phosphorylation and cRel nuclear translocation in LPS-stimulated *Nfkbie*^−/−^ B1a B cells compared to those of their WT counterparts (Fig. 5d, e). No difference was observed between *Nfkbie*^−/−^ and WT B1a B cells in the expression levels of cRel (Fig. 5e) and p65 (not shown) in quiescent cells or after LPS stimulation. These data indicate that IκBε deficiency in CD5+B1a B cells leads to increased NF-κB activation upon stimulation.

Since IκBε deficiency leads to enhanced NF-κB activation, we hypothesized that *Nfkbie*-deficient B1a B cells would be differentially sensitive to NF-κB inhibition. We thus treated *Nfkbie*^−/−^ and WT B1a B cells with increasing concentration of the NF-κB inhibitor IT-901 (ref. ^34^), in presence or absence of LPS stimulation. Cells were analyzed for the growth inhibition and apoptosis induction. We found that IT-901 inhibited cell growth of both *Nfkbie*^−/−^ and WT B1a B cells in a dose-dependent manner; however, the IC50 value was higher for *Nfkbie*^−/−^ B1a B cells (3 µM) compared to that of WT B1a B cells (2.4 μM; Fig. 5f). At 4 µM, no significant difference in cell growth inhibition was observed (Fig. 5f). Analysis of apoptosis showed a significant difference in survival between *Nfkbie*^−/−^ and WT B1a B cells when cells where vehicle treated or IT-901 treated at 0.5 µM, 1 µM, and 2 µM, whereas such difference was lost at 4 µM and 8 µM (Fig. 5g). These data show that *Nfkbie*^−/−^ B1a B cells are generally less sensitive to NF-κB activity inhibition than WT B1a B cells.

Overall these data demonstrate that IκBε is critical to limit B-cell proliferation in response to stimuli through the control of p65 phosphorylation and cRel nuclear translocation. They also establish a link between IgM expression levels and proliferation of *Nfkbie*-deficient B cells.

### *Nfkbie* deficiency enhances GC B-cell proliferation

**Fig. 6:**
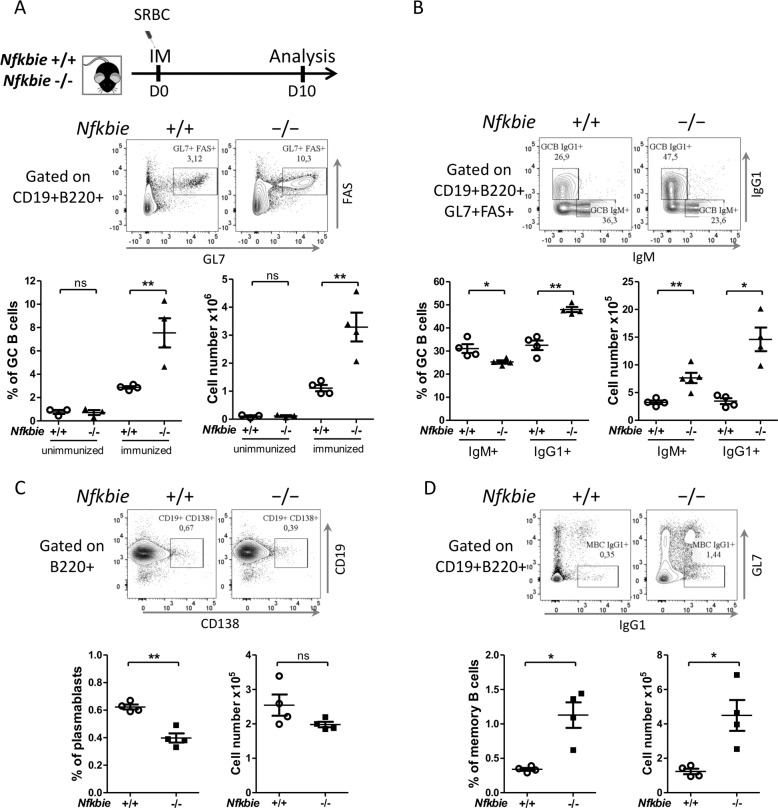
*Nfkbie* deficiency enhances GC B-cell formation. **a** Scheme of the immunization protocol. Mice were intraperitoneally immunized with SRBC on day 0 (D0) and analyzed 10 days afterward (D10). Representative flow cytometry analysis of splenocytes from SRBC-immunized mice of the indicated genotypes. Gates depict germinal center (GC) B cells (B220+CD19+CD95(Fas)+GL7+). Percentages and absolute cell numbers of GC B cells in the spleen of immunized mice are shown. Each symbol represents one mouse (*n* = 4). **b** Flow cytometry plots showing IgM and IgG1 expression by GC (B220+CD19+CD95(Fas)+GL7+) B cells. Histograms show percentages of IgG1+ and IgM+ *Nfkbie*^+/+^, and *Nfkbie*^−/−^ GC B cells. Each symbol represents one mouse (*n* = 4). **c** Splenic plasma cells generation after SRBC immunization of *Nfkbie*^+/+^ and *Nfkbie*^−/−^ mice; shown as FACS profiles (upper panel) and quantitation (lower panel). Each symbol represents one mouse (*n* = 4). **d** Memory B cells formation after SRBC immunization of *Nfkbie*^+/+^ and *Nfkbie*^−/−^ mice; shown as FACS profiles (upper panel) and quantitation (lower panel). Each symbol represents one mouse (*n* = 4). Data are mean ± SEM. *p < 0.05, **p < 0.01; ns, not significant.

GC reaction is necessary for maturation of the humoral immune response, including production of high-affinity plasma cells and memory B-cells. We explored the impact of *Nfkbie* absence in GC by immunizing mice with SRBCs. FACS analysis revealed an increase in both percentages and absolute cell numbers of GC B cells in *Nfkbie*^−/−^ mice compared with those of WT mice (Fig. 6a). Frequency of follicular T-helper cells remained similar in both genotypes (data not shown). In *Nfkbie*^−/−^ mice, IgG1+ GC B cells were significantly increased and IgM+ GC B cells were significantly decreased (Fig. 6b). Percentages but not absolute cell numbers of plasmablasts/plasma cells were reduced in *Nfkbie*^−/−^ mice compared to those of WT mice (Fig. 6c). *Nfkbie*^−/−^ mice had significantly higher percentages and cell numbers of GL7-IgG1+ memory B cells (Fig. 6d). These data suggest that IκBε plays a role in the generation/proliferation of GC B cells, isotype switching, and terminal differentiation of GC B cells.

**Fig. 7:**
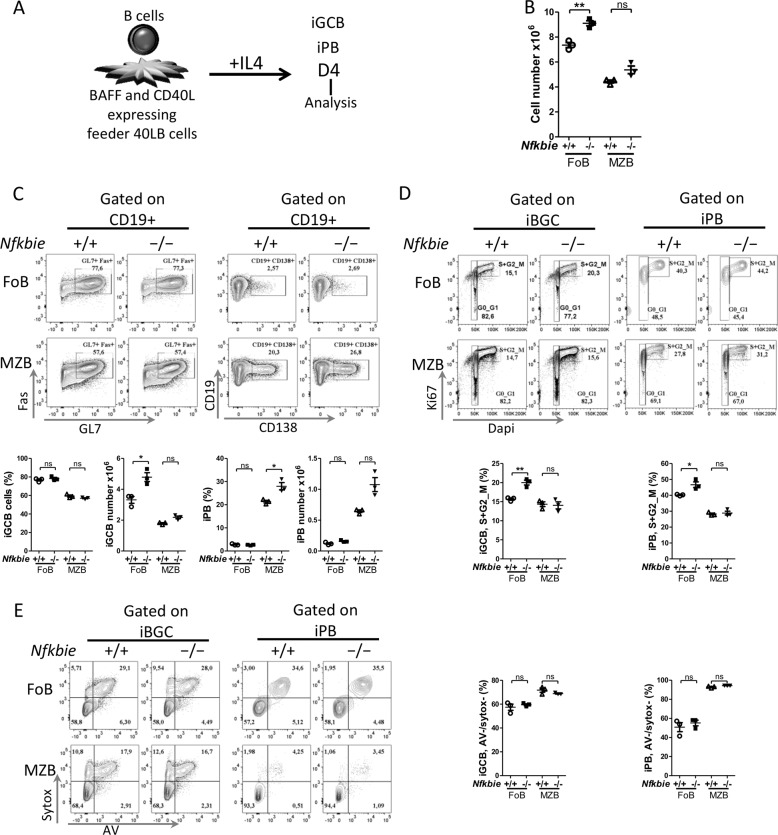
*Nfkbie* deficiency induces germinal center generation and amplification in vitro. **a** Scheme of the germinal center B cells (iGCB) and plasmablasts (iPB) in vitro culture system. **b** Total number of live *Nfkbie*^+/+^ and *Nfkbie*^−/−^ cells cultured on 40LB with IL4 and analyzed on day 4 (D4). Each symbol represents one mouse (*n* = 3). **c** Flow cytometry plots show live iGCB cells (CD19+GL7+FAS+) and iPB (CD19+CD138+) generated from FoB and MZB cells. Histograms present percentages (upper) and absolute cell numbers (lower) of iGCB cells and iPB on day 4. Each symbol represents one mouse (*n* = 3). **d** Representative cell cycle profiles of iGCB cells (left) and iPB cells (right), labeled with Dapi and anti-Ki67, and analyzed by flow cytometry at day 4 (D4). Numbers indicate percentages of cells in the gated populations. Histograms show percentages of the iGCB cells (left) and iPB (right) in the S and G2/M phases of the cell cycle on day 4. Each symbol represents one mouse (*n* = 3). **e** Flow cytometry plots show representative apoptosis analysis of iGCB cells (left) and iPB cells (right), labeled with Annexin V and sytox, and analyzed by flow cytometry at day 4 (D4). Numbers indicate percentages of cells in the gated populations. Histograms show percentages of viable (defined as Annexin V− and sytox−) iGCB cells (left) and iPB (right) on day 4. Each symbol represents one mouse (*n* = 3). Data are mean ± SEM. *p < 0.05, **p < 0.01; ns, not significant.

To establish the cell-autonomous nature of this phenotype, we sorted naive B cells from *Nfkbie*^−/−^ and *Nfkbie*^+/+^ mice, and compared their capacity to generate GC B cells in vitro under culture conditions that mimic the GC reaction^26^ (Fig. 7a for scheme). To avoid the confounding effect of *Nfkbie* deficiency in biasing mature B-cell subsets repartition, we sorted B cells as separate FoB and MZB populations. At day 4 (D4), we observed a significant increase in the total cell numbers of *Nfkbie*^−/−^ FoB cells compared to *Nfkbie*^+/+^ FoB cells (Fig. 7b); however, differences between *Nfkbie*^−/−^ and *Nfkbie*^+/+^ MZB cells did not reach statistical significance (Fig. 7b). Flow cytometry analysis showed significantly increased numbers of in vitro-induced GC B cells (iGCB; CD19+GL7+CD95+) derived from *Nfkbie*^−/−^ vs *Nfkbie*^+/+^ FoB cells but not for MZB cells at D4 (Fig. 7c). In vitro-induced plasmablast (iPB) population (CD19+CD138+) was higher in *Nfkbie*^−/−^ compared to *Nfkbie*^+/+^ MZB cells at D4 (Fig. 7c).

We further investigated iGCB cell number changes in this system by analyzing cell cycle and apoptosis. iGCB cells derived from *Nfkbie*^−/−^ FoB cells displayed a significant increase of S and G2/M phases as compared to iGCB cells derived from *Nfkbie*^+/+^ FoB cells (Fig. 7d). No such difference was observed for MZB cells (Fig. 7d). Analysis of the apoptosis rate showed no difference between *Nfkbie*^−/−^ and *Nfkbie*^+/+^ in both FoB and MZB cells subsets (Fig. 7e).

These in vitro data mirror the in vivo results and indicate that *Nfkbie* deficiency in B cells induces expansion of GC B cells through increasing proliferation of GC B cells in a B-cell-autonomous fashion.

To demonstrate the direct impact of *Nfkbie* deficiency on proliferation via cell cycle, we used a CRISPR/Cas9 approach and successfully generated three knockout Nfkbie clones of the pro-B-cell line Ba/F3 (Supplementary Fig. 6a). We confirmed the knockout of *Nfkbie* gene at the protein level in the three clones (Supplementary Fig. 6a). Proliferation survival and cell cycle analysis were performed in control and edited cells in the presence of IL-3 at 24 h, 48 h, and 72 h. *Nfkbie* knockout clones exhibited increased proliferation when compared to WT cells (Supplementary Fig. 6b). *Nfkbie* knockout clones showed a decrease in the percentages of cells in G0/G1 phase, and an increase in S and G2/M phases as compared to controls (Supplementary Fig. 6c). Analysis of apoptosis failed to detect any changes between knockout and WT cells (Supplementary Fig. 6d). Altogether, these data indicate that the effect of *Nfkbie* deficiency on proliferation of Ba/F3 cells is due to an increased cell cycle entry and is apoptosis independent.

### *Nfkbie* loss cooperates with MYD88 signaling

**Fig. 8:**
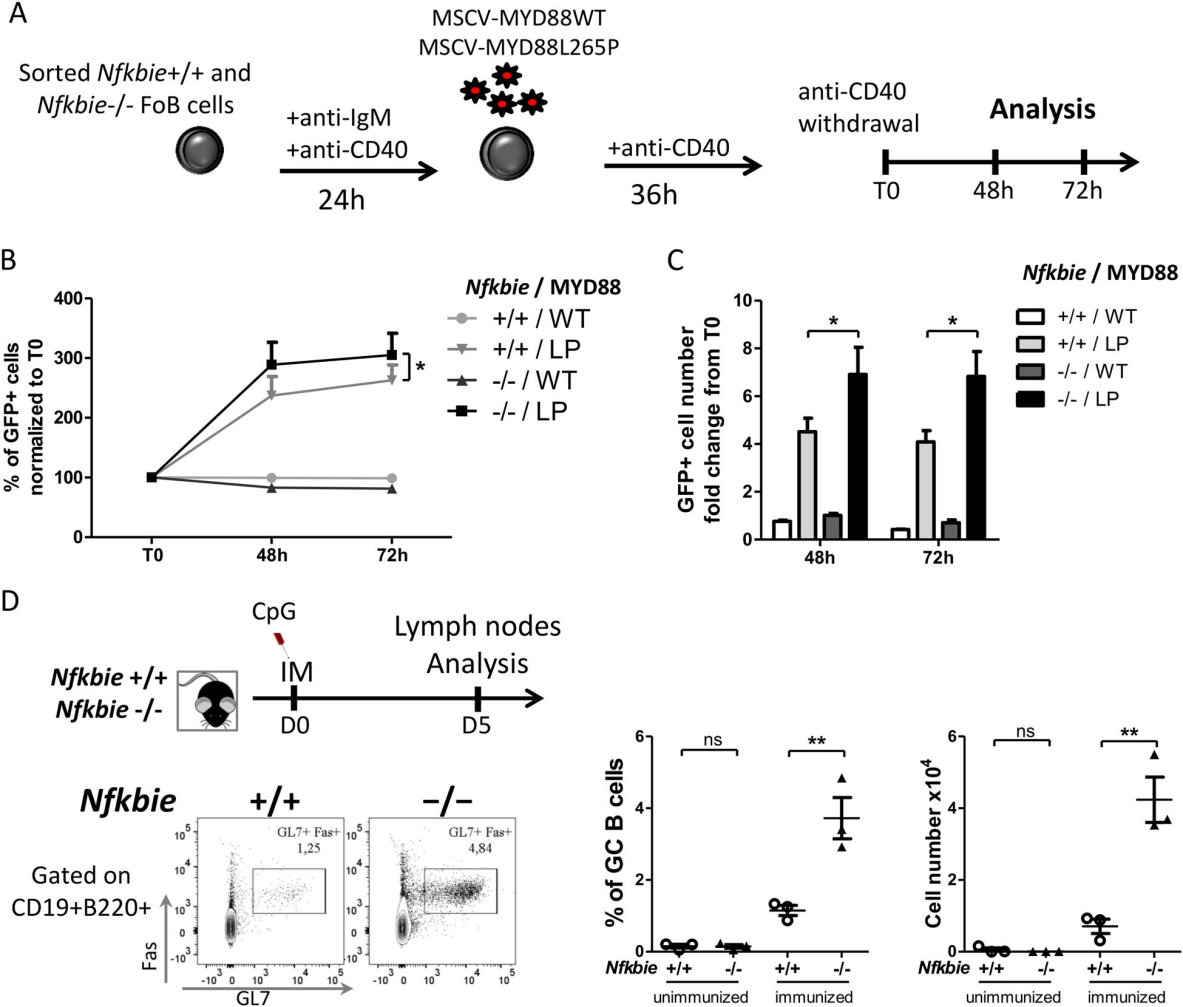
Actively mutated MYD88 cooperate with *Nfkbie* deficiency in B cell. **a** Scheme for retrovirally introducing wild type (WT) and mutant MYD88 (L265P) into activated *Nfkbie*^+/+^ and *Nfkbie*^−/−^ mature splenic B cells. B cells were activated by anti-IgM with anti-CD40 for 24 h. They were then infected with a retrovirus expressing MYD88WT-GFP or MYD88L265P-GFP, and cultured with anti-CD40 for 36 h. Afterward, cells were washed to withdraw anti-CD40 (time (T) 0) and GFP-positive-cell percentages **b** and absolute numbers **c** were monitored at 48 h and 72 h. **b** Percentages of GFP-positive cells normalized to T0. Results are mean ± SD of three independent experiments performed in duplicate. **c** Fold changes of absolute cell numbers of GFP-positive cells normalized to T0. Data are mean ± SD of three independent experiments performed in duplicate. **d** Representative flow cytometry analysis of lymph node from CpG-injected mice of the indicated genotypes. Gates depict germinal center (GC) B cells (B220+CD19+CD95(Fas)+GL7+). The percentage of GC B cells and their cell number in the lymph node are shown. Each symbol represents one mouse (*n* = 3). Data are mean ± SEM. *p < 0.05.

*MYD88* codes for an proximal signaling adaptor downstream of IL-1R and mammalian TLRs. Mutations in *MYD88*, essentially the missense p.L265P mutation, are observed in a wide range of B-cell malignancies^8,10,13,35–39^. They have been shown to activate the JAK and NF-κB pathways^36^. Since both *NFKBIE* and *MYD88* mutations may control TLR signaling and co-occur in human B-cell malignancies (Table 1), we used mutant MYD88 to document the interactions between *MYD88* and *Nfkbie* mutations in primary B cells. Purified WT or *Nfkbie*^−/−^ follicular (CD19+B220+CD23+CD21+) B cells were activated with anti-IgM and anti-CD40, transduced with MYD88 (WT) or MYD88L265P (LP) retrovirus, and cultured with anti-CD40 for 36 h and the proportion of fluorescent cells was monitored in the culture, as described^40^ (Fig. 8a for scheme). As already reported^40^, expression of mutant MYD88 conferred a growth advantage over B cells expressing WT constructs. However, ectopic expression of MYD88LP conferred a significant growth advantage to the *Nfkbie*-deficient-transduced cells in comparison to other genotypes (Fig. 8b). *Nfkbie*-deficient-MYD88LP-transduced cells showed an increased cell number compared to other conditions (Fig. 8c). This indicates that targeting the TLR-NFKB axe endows the double-mutant cell with a growth advantage over the other cells.

**Table 1:** Co-occurrence of NFKBIE and MYD88 mutations in several human B-cell malignancies.

Number of *MYD88*-mutated patient	Number of *NFKBIE*-mutated patient	Number of *MYD88* and *NFKBIE*-mutated patient	B-cell neoplasm	Reference
172	37	5	DLBCL	Reddy et al.^11^
8	4	1	rDLBCL	Morin et al.^17^
1	1	1	PCDLBCL-LT	Fox et al.^37^
15	27	1	CLL	Nadeu et al.^39^
16	1	1	WM	Roos-Weil et al.^35^

*DLBCL* diffuse large B-cell lymphoma, *rDLBCL* relapsed diffuse large B-cell lymphoma, *PCDLBCL-LT* primary cutaneous diffuse large B-cell lymphoma-leg type, *CLL* chronic lymphocytic leukemia, *WM* Waldenström macroglobulinemia.

MYD88 is the canonical adaptor for signaling pathways downstream of the members of the TLR family, including TLR9. Genetic or pharmacological inactivation of TLR9 was shown to inhibits MYD88L265P-induced proliferation in murine and human B-cell lines^40,41^. TLR9 is an essential component of the oncogenic BCR signaling supercomplex (MyD88-TLR9-BCR) identified in ABC-DLBCL and leads to NF-κB activation^41^. Therefore, to examine the cooperation between *Nfkbie* loss and MYD88 signaling in vivo, we immunized mice intraperitoneally with the TLR9 ligand, CpG oligodeoxynucleotides, and analyzed inguinal lymph nodes 5 days later for GC B-cell formation. We observed a significant higher GC B-cell frequency and absolute cell numbers in *Nfkbie*^−/−^ compared to *Nfkbie*^+/+^ mice (Fig. 8d). In the spleen, there was no induction of GC B-cell generation as the percentage of GC B cells was similar to unimmunized mice (data not shown). These data indicate that signaling through TLR9/MYD88 may cooperate with *Nfkbie* deficiency to promote GC B-cell formation and proliferation.

## Discussion

The prevalent inactivation of *NFKBIE* in human BCL and the link between polymorphisms in the *NFKBIE* gene, and human autoimmune and infectious diseases^42,43^ suggest a role for IκBε in B-cell development, function, and transformation. To address this question, we analyzed in detail young and aging *Nfkbie*-deficient mice. Our data indicate that IκBε activity is required for correct B-cell development on the one hand, and for limiting B-cell activation induced by stimuli triggered by pathogens, such as TLR ligands and for restricting proliferation of GC B cells on the other hand. In addition, *Nfkbie* deficiency induces MZB cell amplification —in line with previous observations^20^—due to a higher differentiation of transitional B cells toward MZB cells over the FoB-cell pathway. In B cells, IκBε provides negative regulation upon TLR stimulation by controlling NF-κB activation through limiting nuclear translocation of Rel-containing NF-κB dimers (p65 and cRel)^2,20–22^. In LPS-stimulated IκBε-deficient CD5+B1a B cells, we observed stronger p65 phosphorylation and cRel nuclear translocation, suggesting enhanced NF-κB activity in these cells. This was further supported by NF-κB activity inhibition experiments, which uncovered a decreased sensitivity to drug-induced apoptosis and cell proliferation inhibition of IκBε-deficient B1a B cells, with respect to their WT counterparts.

NF-κB activity downstream BCR and cytokine receptors signaling plays critical roles in the commitment of transitional B cells into follicular or MZB cell fate^29^ and in the development of mature B1 B cells^28,30^. Mice deficient in components of the alternative NF-κB pathway (e.g., NF-κB2) or of the classical NF-κB pathway (e.g., NF-κB1 and Rel), present a drastic reduction in the number of FoB and MZB cells, and B1 B cells, respectively^28^. In keeping with its negative regulator function^20,21^, absence of *Nfkbie* elicits elevated NF-κB activity downstream BCR and cytokine receptors^2,20–22^ that likely accounts for the bias in mature B-cell development observed here.

We also uncovered that *Nfkbie* activity is required for correct development and function of B1 B cells. B1 cells might differentiate from fetal liver and adult bone marrow B1 progenitors^31^. The bone marrow of *Nfkbie*^−/−^ mice contains more B1 progenitors than WT mice. However, we did not observe increased proliferation or survival of the B1 B cells directly isolated from *Nfkbie*^−/−^ and WT mice. These findings suggest a role for IκBε in the generation or expansion of B1 progenitor pool. BCR signaling plays an instructive role in B1a cell lineage determination and maintenance^28,30,44,45^. In addition, recent studies showed that BCR signaling cooperates with TLR signaling in controlling expansion and activation of B1a B cells^46–49^. NF-κB is one of the key transcription factors activated by BCR and TLR signaling. Consequently, increase of NF-κB activation upon BCR and TLR signaling likely drives the expansion of B1a cell compartment observed in the *Nfkbie*-deficient mice.

Consistent with IκBε being required to limit B-cell activation^20,22^, our findings revealed that *Nfkbie* deficiency enhances proliferation of MZB and B1a IgMhi B cells in response to the TLR4 and TLR9 ligand, LPS, and CpG oligodeoxynucleotides respectively. B1a cells of *Nfkbie*-deficient mice show a significant downregulation of IgM expression in peritoneal B1a cells, with respect to WT and presented two B220lowCD19+CD5+B1a populations with different levels of IgM expression (B1a IgMhi and B1a IgMlow). *Nfkbie*^−/−^ B1a IgMlow population showed impaired proliferation response to TLRs stimuli and reduced ex vivo survival compared to B1a IgMhi cells, in agreement with the key role of BCR in B1 cells expansion and activation^28,30,45–49^. Downregulation of IgM on B cells contributes to a physiological B-cell immune tolerance mechanism called anergy, and serves to restrain their response to endogenous/self-antigens and to chronic BCR stimulation^50^. Our results strongly suggest that the B1a IgMlow population is phenotypically and functionally anergic as defined by in vivo downregulation of surface IgM expression, CD5 expression, in vitro hyporesponsiveness to TLR-dependent stimuli and ex vivo-reduced survival^50–54^. Importantly, these properties are hallmark features of human CLL^44,54^, in which *NFKBIE* is mutated in ~10% of cases^2,3,15,16^. Recent work has uncovered cooperation between BCR and TLR in response to innate mitogenic stimuli. BCR-deficient B cells show impaired proliferation in response to LPS or CpG, indicating that correct BCR surface expression and signaling are critical for B-cell proliferation in response to such innate immune stimuli^48,49^. However, whether regaining IgM surface expression reverses anergic functions in these cells, as reported for autoreactive anergic cell model studies^55^, needs to be tested.

*Nfkbie*-deficient mice develop several features of human lymphoproliferative B-cell disorders, in particular, MBL, a low-penetrance premalignant stage^13,56^. Firstly, *Nfkbie* deficiency in mice leads to the expansion of an oligoclonal CD19+CD5+B-cell subset, and in humans, mature CD19+CD5+B cells, show high transcriptional and functional similarity to CLL B cells and are candidate for being a normal cellular counterpart of CLL B cells^13,45,54^. Secondly, *Nfkbie*-deficient B1a cells exhibited increased survival, proliferation, and enhanced NF-κB activity in response to stimulation consistently with the observations made for *NFKBIE*-mutated human CLL B-cells^2^. Thirdly, B220lowCD19+CD5+B cells expanded dramatically in the peripheral blood of old *Nfkbie*-deficient mice and infiltrated several organs, including spleen and lymph nodes. Finally, the lymphoproliferative disease developed by *Nfkbie*-deficient mice is indolent and has low penetrance, resembling the mostly indolent course of MBL and CLL, diseases of elderly population. Aging is associated with activation of innate immune cells, including a subset of B1a B cells, due to the increased auto- and pathogen-derived antigen availability, age-associated immune dysfunction, and age-induced gut permeability^45,57,58^. These age-associated changes could participate in the generation of MBL-like and/or CLL-like malignancies^45,54,59^. In this perspective, the indolent MBL development in *Nfkbie*^−/−^ mice might largely be attributed to the increased survival and hyperproliferation of B1a IgMhi B cells, in response to immune stimulation that would be more available with age and the anergic nature of B1a IgMlow B cells and their hyporesponsiveness to stimulation. Indeed, in mice, MBL and CLL develops from B1a CD5+B cells and several study demonstrate a crucial role for BCR expression, reactivity, and signaling in the malignant transformation of these cells^45,60–62^.

NF-κB activity plays an important role in the regulation of GC reaction^32,63^. Conditional deletion of Rel and Rela in GC B cells revealed that Rel is required to maintain GC populations, whereas Rela promoted Blimp-1-mediated plasma cell development^63^. Our data show that under T-cell-dependent immunization condition, *Nfkbie* deficiency promotes the excessive generation of GC B cells but not TFH cells in vivo. The amplification of GC B cells seems to be caused by an increased proliferation of *Nfkbie*-deficient GC B cells, suggesting a role for IκBε in controlling proliferation of GC B cells. In line with these findings, it was recently shown that activating mutations in CARD11 (observed in ~5% of DLBCL (ref. ^5^)), a NF-κB activator protein downstream BCR signaling, increases GC formation and GC B-cell proliferation through enhanced cycling^64^. GC B cells represent the origin of several BCLs including DLBCL (ref. ^32^). Deregulated NF-κB signaling is a hallmark of GC-derived B-cell malignancies^3,5,6,8^. Mutations in *NFKBIE* gene are detected in ~5% of DLBCL and 23% of PMBCL (refs. ^12,17^). Our results provide a functional link between *Nfkbie* mutations and GC-derived BCL. As pointed above, the absence of *Nfkbie* induces a bias of B2 B-cell development toward MZB cells. MZB B cells are the normal cellular counterpart of splenic marginal zone lymphoma, a BCL in which *NFKBIE* is also mutated in ~3% of cases^12^. Our data strongly suggest that IκBε plays an essential role in regulating proliferation and differentiation of the B cells from which these lymphomas derive.

In CLL and several BCL, aberrant NF-κB activation is the result of genetic alterations in pathways leading to NF-κB activation and aberrant stimulation from the microenvironment (through BCR, CD40, and TLR)^2,3,5,6,8,10–12,32^. In vivo injection of the TLR9 ligand, CpG oligodeoxynucleotide, increased generation, and proliferation of *Nfkbie*^−/−^ GC B cells compared to that of WT ones. Accordingly, MYD88L265P mutation, a mutation observed across many subtypes of human lymphoid malignancies, increased proliferation of *Nfkbie*^−/−^ cells compared to that of WT B cells in vitro. In line with these findings, in Waldenström macroglobulinemia and DLBCL human cell lines, inactivation of TNFAIP3 (encoding A20, a negative regulator of NF-κB signaling) also cooperates with MYD88LP mutation to enhance NF-κB activation and resistance to BCR signaling inhibitor, ibrutinib^65,66^. Together, these observations demonstrate that inactivation of *Nfkbie* in mice predisposes to MBL and CLL, and upon collaboration with oncogenic events, it might promote BCL development.

## Supplementary information

Supplementary_Figures 1–6 and legendes

Supplementary Methods
