# Supplementary material for: Nfkbie-deficiency leads to increased susceptibility to develop B-cell lymphoproliferative disorders in aged mice
Source: Blood Cancer J. 2020 Mar 13;10(3):38. doi: 10.1038/s41408-020-0305-6 (PMC7070037; doi:10.1038/s41408-020-0305-6)
Supplement: Supplementary file 1 — Supplementary_Figures 1–6 and legendes [file 41408_2020_305_MOESM1_ESM.pdf]

Supplementary Figure1

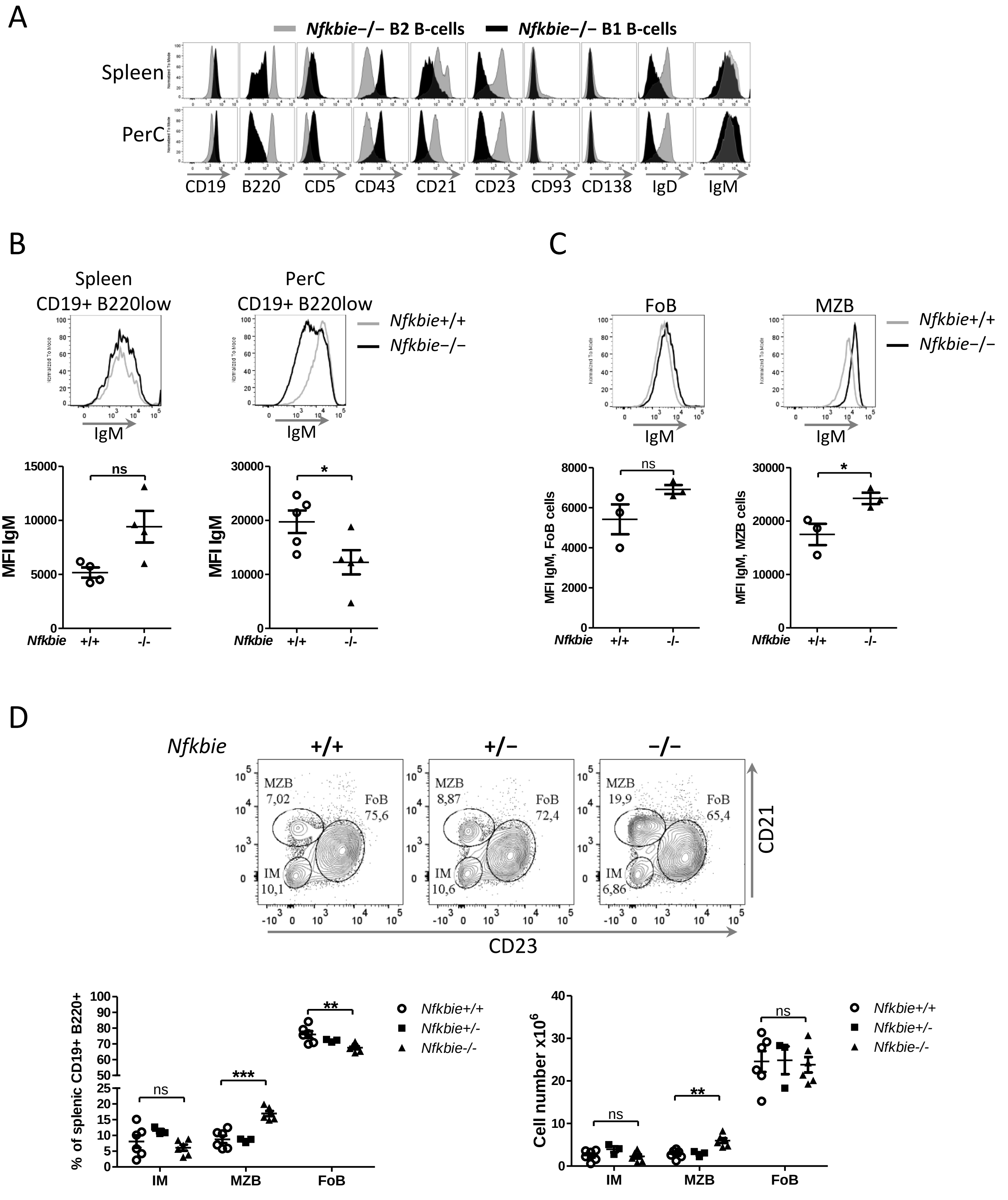

Supplementary Figure1. Analysis of IgM expression on aging *Nfkbie*−/− B cells subsets

A. Representative flow cytometry profiles depicting the immunophenotype of B2 B-cells (CD19+B220+, gray) and B1 B-cells (CD19+B220low, black) in spleen (n=6) and PerC (n=4) of a *Nfkbie*−/− mouse.

B. IgM expression on splenic and PerC CD19+ B220low B cells of *Nfkbie*+/+ and *Nfkbie*−/− mice. Each symbol represents one mouse. MFI: Mean fluorescence intensity.

C. IgM expression on splenic follicular (FoB) and marginal zone (MZB) B cells of 8-9 month-old *Nfkbie*+/+ and *Nfkbie*−/−mice. Each symbol represents one mouse. MFI: Mean fluorescence intensity.

D. Representative FACS profiles of splenic B2 (CD19+B220+) B-cell populations. CD23 and CD21 expression in the B220+CD19+ gate showing the Follicular (FoB: CD23+CD21+), Marginal zone (MZB: CD23lowCD21high) and immature (IM: CD23-CD21-) B cells. Percentages and absolute cell numbers of splenic MZ and FoB cells are shown. Each symbol represents one mouse.

Data are mean ± SEM. *: $p < 0.05$, **: $p < 0.01$, ns : not significant.

Supplementary Figure2

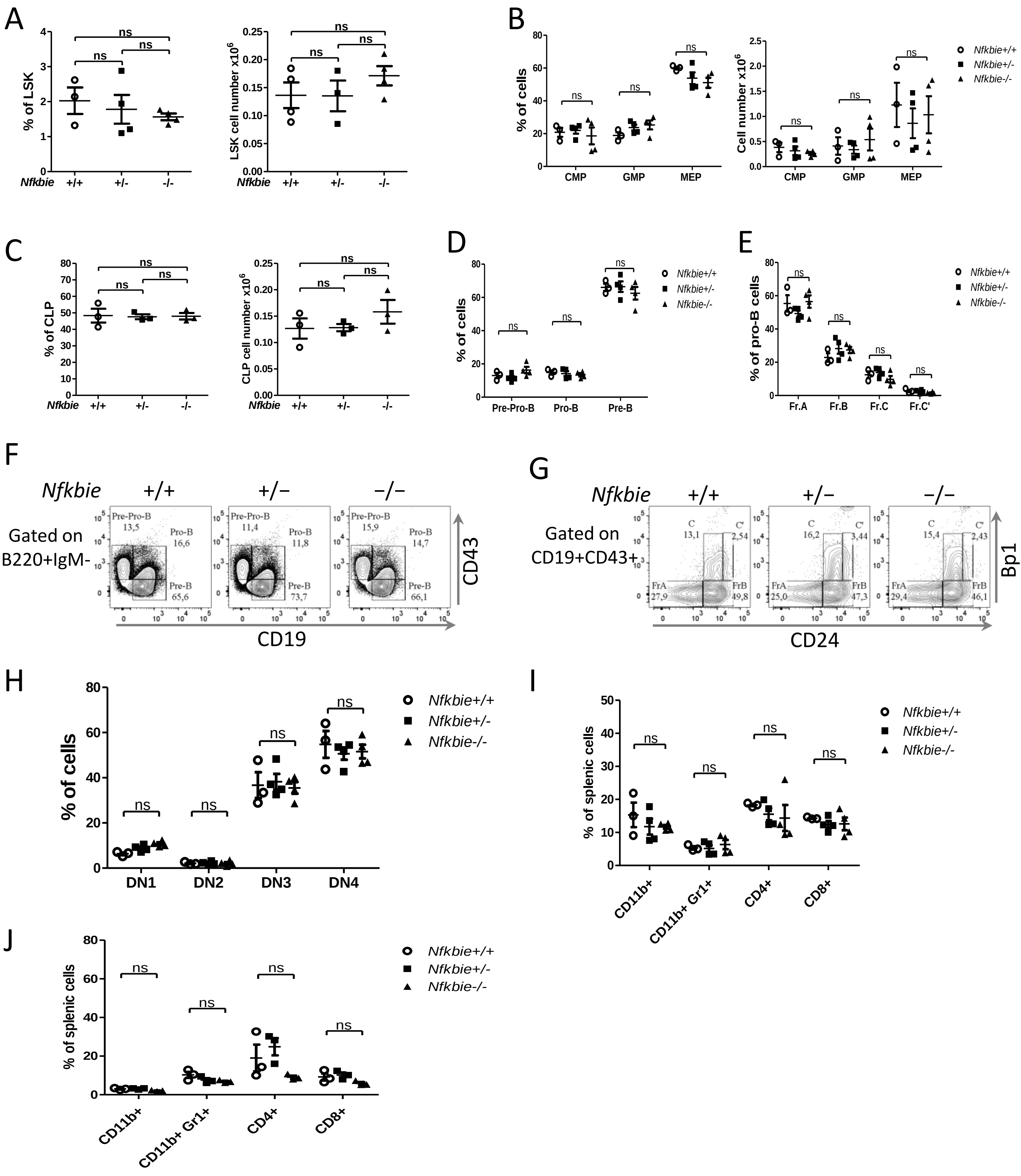

Supplementary Figure2. Analysis of hematopoietic lineage in *Nfkbie*−/− mice

A. Proportions and absolute cell numbers of LSK cells in the bone marrow (BM) of 2-month-old *Nfkbie*+/+, *Nfkbie*+/− and *Nfkbie*−/− mice. Each symbol represents one mouse.

B. Proportions and absolute cell number of each of the myeloid progenitors (CMP, GMP, and MEP) in the bone marrow of *Nfkbie*+/+, *Nfkbie*+/− and *Nfkbie*−/− 2-month-old mice. Each symbol represents one mouse.

C. Proportions and absolute cell numbers of common lymphoid progenitor (CLP) cells in the bone marrow of 2-month-old *Nfkbie*+/+, *Nfkbie*+/− and *Nfkbie*−/− mice. Each symbol represents one mouse.

D. Early B-cell development analysis of bone marrow from 2-month-old mice. Total BM cells were analyzed for B220 and IgM expression. Then CD19 and CD43 expression was evaluated in gated B220+IgM- cells to define pre-pro-B (CD19-CD43+), pre-B (CD19+CD43-) and pro-B (CD19+CD43+) populations. Each symbol represents one mouse.

E. The more immature Hardy fractions (Fr.A CD24-Bp1-; Fr.B: CD24+Bp1-; Fr.C: CD24+Bp1+ and Fr.C': CD24hiBp1+) were analyzed in the pro-B-cell gate. Each symbol represents one mouse.

F. Representative flow cytometry profiles of early B-cell development analysis in the bone marrow of 2-month-old mice. Total BM cells were analyzed for B220 and IgM expression. Then CD19 and CD43 expression was evaluated in gated B220+IgM- cells to define Pre-pro-B (CD19-CD43+), pre-B (CD19+CD43-) and pro-B (CD19+CD43+) populations.

G. Representative flow cytometry profiles of the more immature Hardy fractions (Fr.A CD24-Bp1-; Fr.B: CD24+Bp1-; Fr.C: CD24+Bp1+ and and Fr.C': CD24hiBp1+) analyzed in the pro-B-cell gate.

H. Percentages of DN1 (CD44+CD25-), DN2 (CD44+ CD25+), DN3 (CD44-CD25+) and DN4 (CD44-CD25-) cells in the thymus of 2-month-old *Nfkbie*+/+, *Nfkbie*+/− and *Nfkbie*−/− mice. Each symbol represents one mouse.

I. Percentages of CD11b+, CD11b+Gr1+, CD4+ and CD8+ cells in the spleen of 2-month-old *Nfkbie*+/+, *Nfkbie*+/− and *Nfkbie*−/− mice. Each symbol represents one mouse.

J. Percentages of CD11b+, CD11b+Gr1+, CD4+ and CD8+ cells in the spleen of 5-7-month-old *Nfkbie*+/+, *Nfkbie*+/− and *Nfkbie*−/− mice. Each symbol represents one mouse.

Data are mean ± SEM. ns : not significant.

Supplementary Figure 3

A

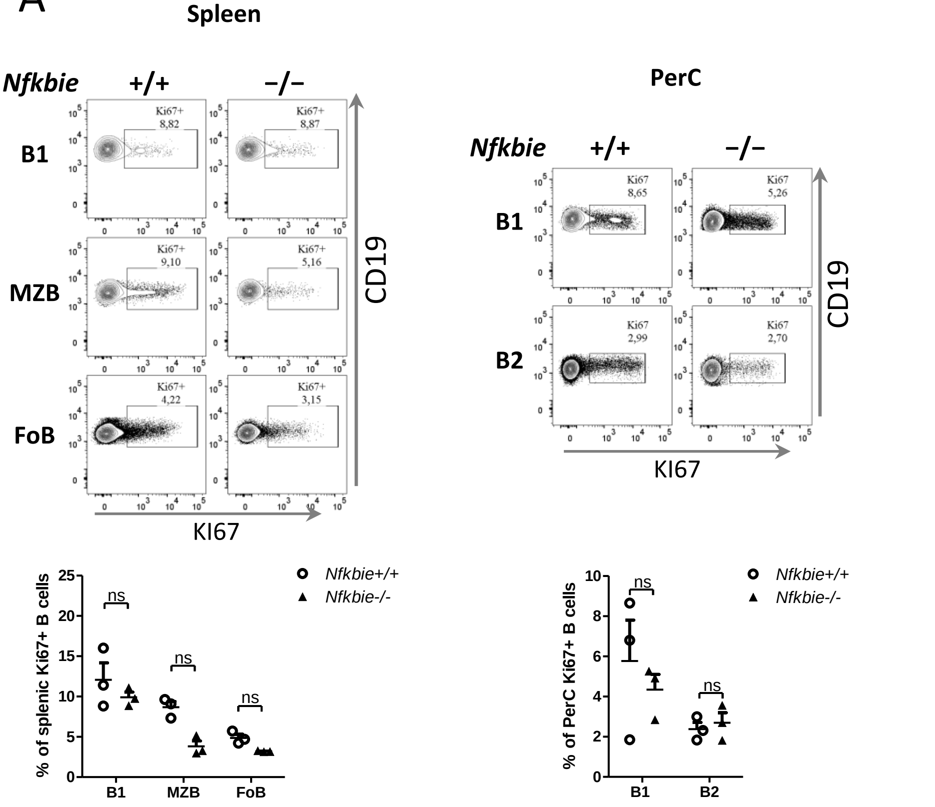

B

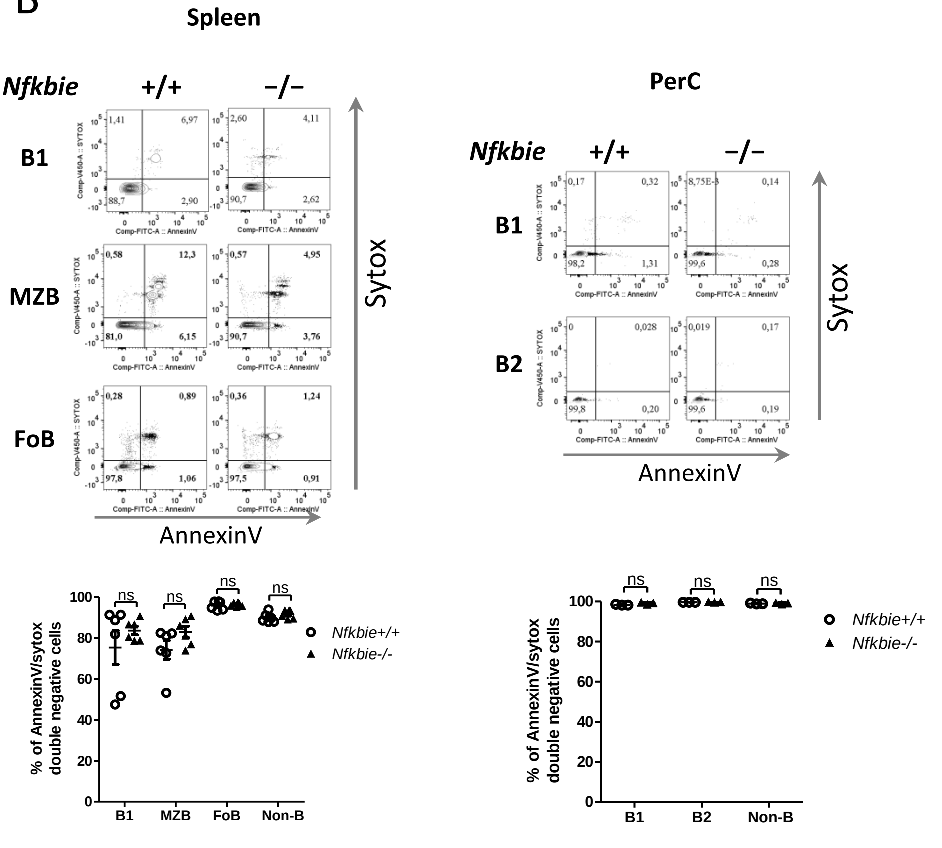

Supplementary Figure 3. ***Nfkbie*-deficiency effect on B cell subset survival and proliferation.**

A. Left panel: Representative flow cytometry profiles and percentages of splenic B1 B-cells, Marginal zone B cells (MZB) and Follicular B cells (FoB) expressing the ki67 marker. Right panel: Representative flow cytometry profiles and percentages of peritoneal B1 and B2 B cells expressing the ki67 marker (PerC). Each symbol represents one mouse.

B. Viability of B cells directly isolated from the spleen (n = 6) and the peritoneal cavity (PerC) (n = 3) of *Nfkbie*+/+ and *Nfkbie*-/- mice defined as Annexin V− and sytox− by flow cytometry. Cells were immediately stained after isolation with anti-CD19, -B220, -CD21, -CD23 and Annexin V and Sytox. Non-B cells are defined as CD19- B220-. Left panel: Representative flow cytometry profiles and percentages of viable splenic B1 B-cells, Marginal zone B cells (MZB) and Follicular B cells (FoB). Right panel: Representative flow cytometry profiles and percentages of viable peritoneal B1 and B2 B cells (PerC).

Data are mean ± SEM. *P < 0.05, ns : not significant.

Supplementary Figure4

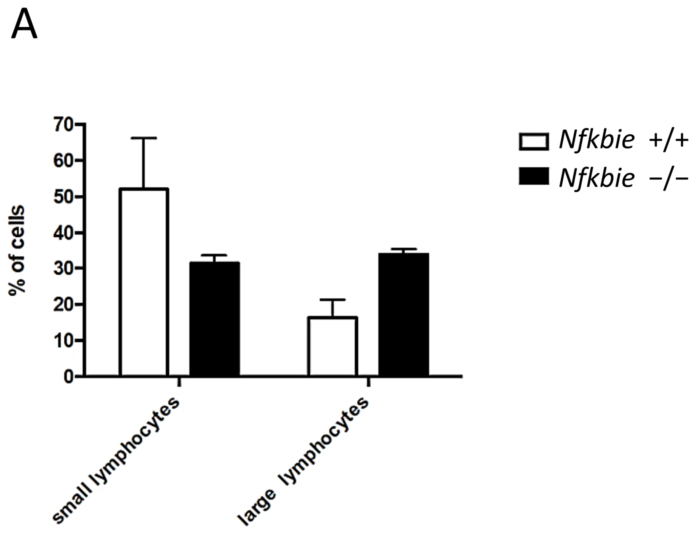

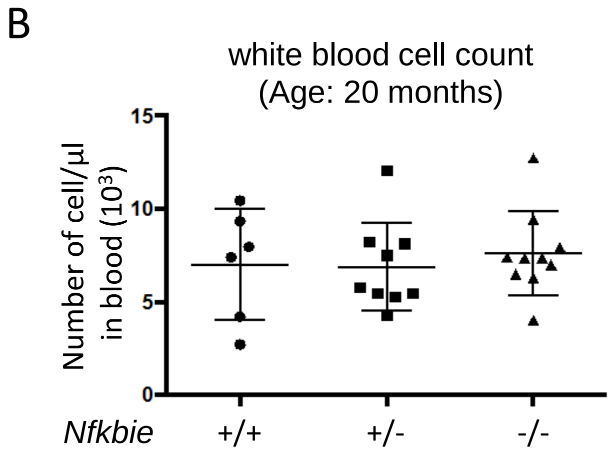

Supplementary Figure4. Blood cell analysis

A. Cytological analysis of blood smears of 19-month-old *Nfkbie*+/+ (n=2) and *Nfkbie*−/− (n=2) mice.

B. White blood cell count of 20-month-old *Nfkbie*+/+, *Nfkbie*+/− and *Nfkbie*−/− mice.

Supplementary Figure 5

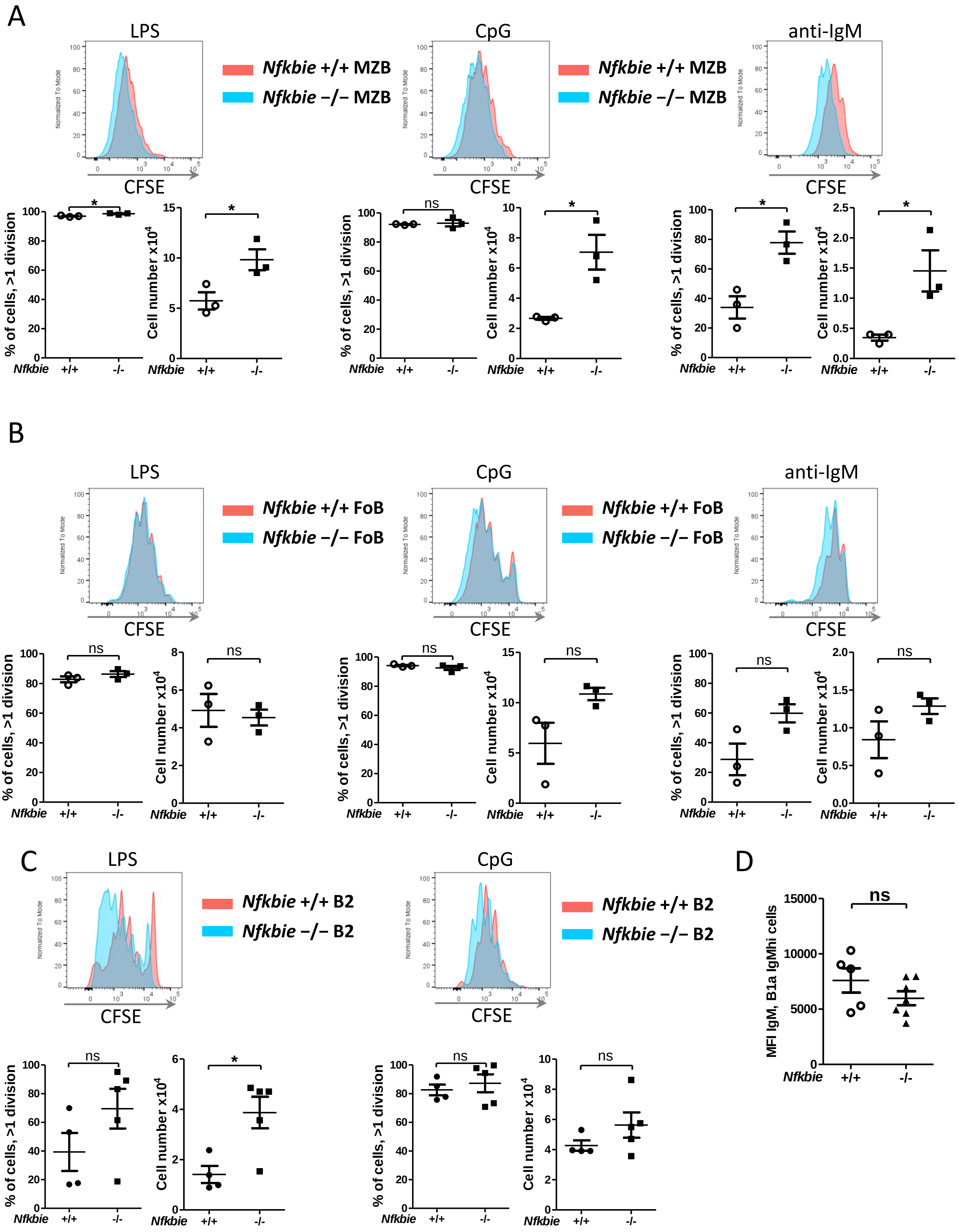

Supplementary Figure 5. *Nfkbie*-deficient B cell subsets proliferation in response to TLR and BCR stimulations

A. Sorted splenic marginal zone B-cells (MZB: CD19+ B220+ CD23low CD21high) were labeled with CFSE and cultured in presence of LPS, CpG or anti-IgM. Cell division was measured by CFSE dilution at 72 h. Percentages of cells that underwent at least one division and absolute cell numbers at 72 h are shown. Each symbol represents one mouse.

B. Sorted splenic follicular B-cells (FoB: CD19+ B220+ CD23+ CD21+) were labeled with CFSE and cultured in presence of LPS, CpG or anti-IgM. Cell division was measured by CFSE dilution at 72 h. Percentages of cells that underwent at least one division and absolute cell numbers at 72 h are shown. Each symbol represents one mouse.

C. Sorted peritoneal B2 B-cells (B2: CD19+ B220+) were labeled with CFSE and cultured in presence of LPS or CpG. Cell division was measured by CFSE dilution at 72 h. Percentages of cells that underwent at least one division and absolute cell numbers at 72 h are shown. Each symbol represents one mouse.

D. IgM expression levels on peritoneal B1a (CD19+ B220low CD5+) B cells of *Nfkbie*+/+ and B1a IgMhi (CD19+ B220low CD5+ IgMhi) *Nfkbie*−/−mice. Each symbol represents one mouse. MFI: Mean fluorescence intensity.

Data are mean ± SEM. *: $p < 0.05$, ns: not significant.

Supplementary Figure 6

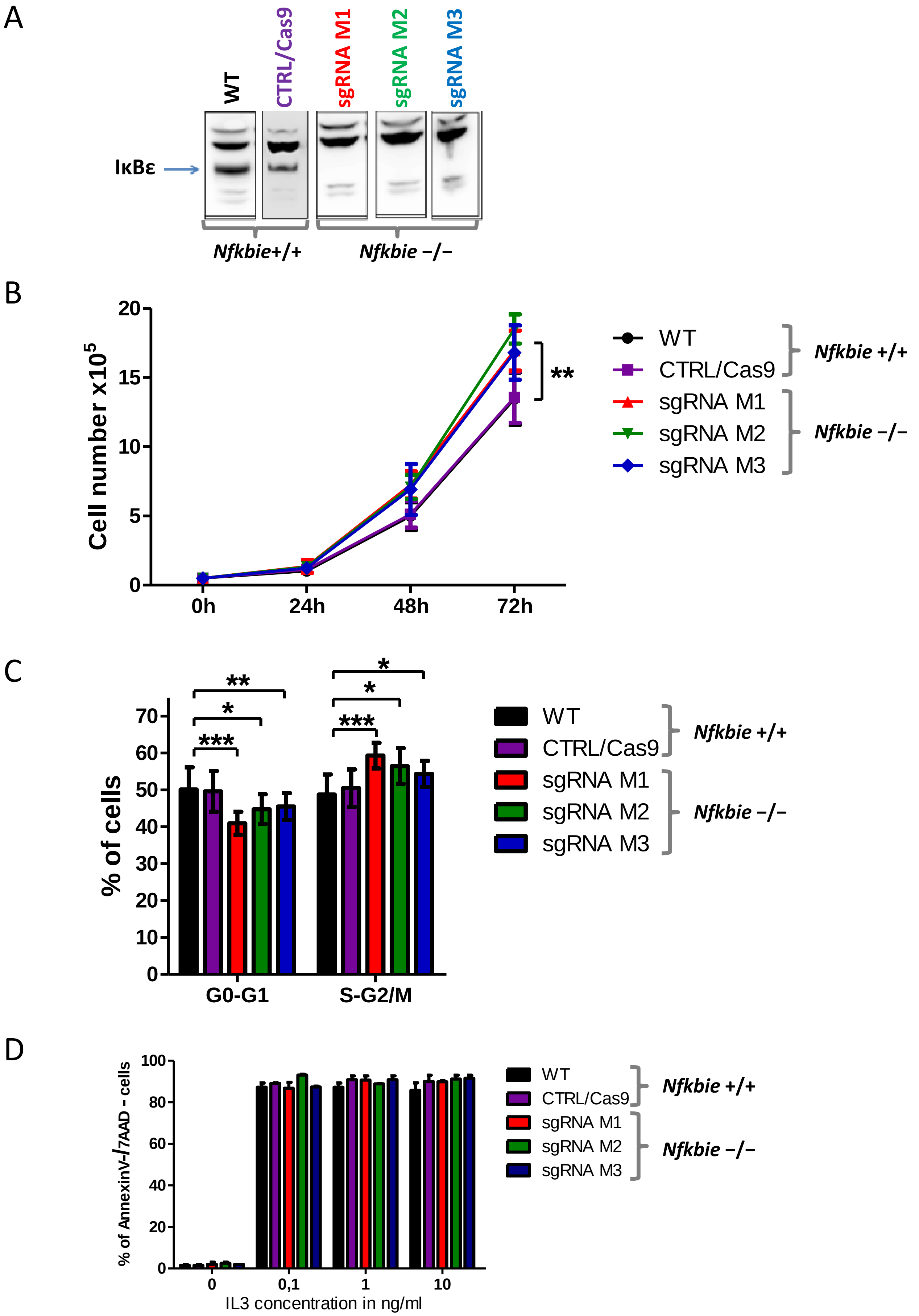

Supplementary Figure 6. Impact of *Nfkbie*-deficiency on cell proliferation, cell cycle progression and apoptosis in Ba/F3 cell line.

A. Western blot analysis of IκBε expression in the Ba/f3 parental cell line (WT), the *Nfkbie*+/+ Ba/f3 clone (transfected with a vector containing the Cas9 gene without sgRNA, CTRL/Cas9) and three *Nfkbie*−/− clones using three different sgRNA guides (sgRNA M1, M2, M3).

B. $2x10^6$ cells/well were cultured in 12-well plates in 1 ml medium in presence of 10ng/ml of rmIL-3. Changes in viable cell numbers were assessed by a trypan blue exclusion method after 24, 48 and 72 hours of culture (n= 3 independent experiments performed in triplicate).

C. DNA content of living cells was measured by propidium iodide staining after 48h of culture. Results are represented as the summed percentages of cells in the G0/G1 and S-G2/M phases of the cell cycle (n= 3 independent experiments performed in triplicate).

D. Apoptosis was evaluated after 48 hours of culture by annexin V–PE/7-AAD staining in the presence of different concentration of rmIL3. Percentages of double-negative (i.e. annexin-V-negative and 7AAD-negative) living cells are shown (n= 3 independent experiments performed in triplicate).

Data are mean ± SEM. *: $p < 0.05$, **: $p < 0.01$, ***: $p < 0.001$
