# Supplementary material for: Nfkbie-deficiency leads to increased susceptibility to develop B-cell lymphoproliferative disorders in aged mice
Source: Blood Cancer J. 2020 Mar 13;10(3):38. doi: 10.1038/s41408-020-0305-6 (PMC7070037; doi:10.1038/s41408-020-0305-6)
Supplement: Supplementary file 2 — Supplementary Methods [file 41408_2020_305_MOESM2_ESM.docx]

**Flow Cytometry**

Total white blood cells, obtained from peripheral blood and single-cell suspensions from bone marrow, spleen, lymph nodes and thymus were stained after ammonium chloride lysis of red blood cells, in PBS (Invitrogen) supplemented with 2% fetal bovine serum (FBS). The following anti-mouse antibodies were purchased from BD Bioscience™ or Ebioscience™: CD3 (145-2C11), CD4 (RM4-5), CD5 (53-7.3), CD8 (53-6.7), CD11b/Mac1 (M1/70), CD19 (1D3), CD21/35 (4E3), CD23 (B3B4), CD24 (M1/69), CD43 (R2/60), CD117/Kit (ACK2), CD127/IL7Rα, CD21/35 (4E3), CD23 (B3B4), CD24, IgD (11-26c.2a), IgM (AF6-78), TER119 (TER-119), NK1.1 (PK136), Bp1 (Bp1) CD93 (AA4.1), CD16/32 (2.4G2), Sca (D7), CD138 (281-2) and GL7 (GL7). Anti-cRel (1RELAH5) antibody was purchased from eBioscience and anti-Phospho-NF-κB p65 (Ser536) (93H1) was purchased from Cell Signaling Technology. mAbs were conjugated with fluorescein isothiocyanate (FITC), phycoerythrin (PE), PerCP-Cyanin (Cy)5.5, PeCy7, allophycocyanin (APC), APC-Cy7, Pacific blue (Pb), APCeFluor780 or brilliant violet 510 (BV510). Flow cytometric analysis was performed using a FACS CantoII Flow Cytometer (BD Biosciences) and cell sorting using a FACSAriaIII or Influx (BD Biosciences). Immunophenotypic data were analyzed using the FlowJo Version 10 software (TreeStar, Inc.).

For cRel intracellular staining, cells were fixed and permeabilized using the Transcription Factor Buffer Set (BD Biosciences) following manufacturer’s instructions. Intranuclear cRel staining was performed as previously described ^33,34^.

For phospho-flow, cells were fixed with BD Cytofix™ Fixation Buffer (BD Biosciences) and then permeabilized with BD Phosflow Perm Buffer II (BD Biosciences) following manufacturer’s instructions.

The gating of cells was based on the isotype control and fluorochrome minus one (FMO) settings.

**Cell isolation and culture**

For B1a cell isolation, after ammonium chloride lysis of red blood cells, homogenized splenocytes or cells from peritoneal lavage were incubated with anti-CD3, anti-Gr1, anti-NK1.1 (dump channel), and anti-B220, anti-CD19, anti-CD5 and anti-IgM at 4°C. Following this incubation, cells were washed with PBS containing 2% FBS and sorted using FACSAriaIII cell sorter (BD Biosciences). B1a B-cells were sorted as Dump-CD19+B220lowCD5+. For experiments in which separation of splenic B1 B-cells, marginal zone (MZ) B-cells and follicular (FO) B-cells was performed, splenocytes were incubated with anti-CD3/anti-Gr1/anti-NK1.1 (dump channel), anti-B220, anti-CD19, anti-CD23 and anti-CD21 and B-cell population separation was performed using a FACSAria III cell sorter (BD Biosciences). B1 B-cells were sorted as Dump-CD19+B220low, MZ B-cells were sorted as Dump-CD19+B220+CD23+CD21hi and FO B-cells as Dump-CD19+B220+CD23hiCD21+. Cells were cultured in RPMI 1640 (Invitrogen) supplemented with 10% FBS, 10 mM HEPES, 1 mM sodium pyruvate, 1 mM nonessential amino acids, 0.055 mM 2-ME, 100 U Penicillin/Streptomycin and 0.3 mg/ml glutamine (all from Invitrogen). B-cells were stimulated with either 10 µg/ml F(ab′)2 anti-IgM (Jackson ImmunoResearch) or 10 µg/ml LPS (Sigma) or 1.5 µM CpG (Invivogen).

For NF-κB activity inhibition, cells were treated with DMSO (vehicle) or with serial dilutions (0.5 µM to 8 µM) of IT-901 (TOCRIS). The half-maximal inhibitory concentration (IC50) is defined as the concentration of IT-901 that decreases cell growth by 50% in treated cells compared to control cells at 72h.

**Flow cytometry analysis of cell proliferation, survival and cell cycle**

For proliferation assays, 2.5–3 x10^4^ purified B-cells were stained with 5 µM CFSE (Invitrogen) and cultured in complete media with previously mentioned stimuli (see above). B-cells were collected at 72h and stained with sytox (Life technologies) to exclude dead cells. CFSE dilution assessed as the percentage of cells that achieved at least one division. B-cells were analyzed for proliferation and survival using a FACS CantoII flow cytometer (BD Biosciences). Cell death was evaluated using sytox staining at indicated time of culture. Apoptosis was measured by flow cytometry using annexin-V–phycoerythrin (PE) and 7-amino-actinomycin (7-AAD) (BD Biosciences) or sytox blue staining according to the manufacturer’s recommendations.

For cell cycle analysis of BaF3 cell line, 1x10^6^ cells were washed with cold PBS and then fixed with dribs and drabs ice cold 70% ethanol under gently vortexing. Fixed cells were then centrifuged and washed twice with PBS and treated with 100 µL PB RNase (100 µg/mL) for 30 min. Cells were stained with 250 µL of propidium iodide (50 µg/mL) for further 30 min in the dark. For cell cycle analysis of primary B-cells, 1x10^6^ cells were fixed and permeabilized using Transcription Factor Buffer Set (BD Biosciences) according to the manufacturer’s recommendations. Cells were first labeled with anti-Ki67-Alexa Fluor 647 (BD Biosciences) and then with sytox blue. DNA content was analyzed after exclusion of aggregates using FACS cantoII flow cytometer (BD Biosciences).

**Competitive bone marrow transplantation assay**

10 x 10^3^ LSK cells from 2- to 3-month-old WT, *Nfkbie*+/− and *Nfkbie*−/− CD45.2+ mice along with 2.5 x 10^6^ total bone marrow cells from WT CD45.2+ CD45.1+ mice, were transplanted into lethally irradiated CD45.1+ recipient mice. Five weeks after transplantation, the recipients were assessed for CD45.2/CD45.1 chimeras in their peripheral blood by FACS analysis. The contribution of CD45.1+ CD45.2+ versus CD45.2+ cells in the peripheral blood was monitored every 5-7 weeks until 22 weeks.

**CRISPR *Nfkbie* knockout in Ba/F3 cell line**

sgRNA and Cas9 were produced from a single CRISPR-GFP lentiviral plasmid (kindly provided by Julie Rivière). Three CRISPR guide sequences were designed to target the first exon common to all murine *Nfkbie* isoforms using <http://crispr.mit.edu/>; sgRNA1 GGCTCCGGATCATCCCAAAGCGG, sgRNA2 AGATGCGGATGGAGAGCGAGCGG, sgRNA3 ACAGCCTTGGAGACATGCTCCGG. Transient nucleofection of 2.5 × 10^6^ Ba/F3 cells with 8 µg of plasmid DNA was performed by using the Amaxa Nucleofector device (Lonza) according to the supplier’s recommendations. Single GFP+ cells were FACS-sorted in 96-well plates 48 hours later and analyzed by Sanger sequencing of PCR encompassing the targeted site. Three clones for each sgRNA guides were selected and checked for the absence of the IκBε protein by western blot. Parental Ba/F3 WT cells and cell transfected with CRISPR vector expressing Cas9 but not RNA guide were used as control. Ba/F3 cell line was authenticated by IL3-dependency and were negative for mycoplasma.
